# Targeting SARS-CoV-2 Proteases for COVID-19 Antiviral Development

**DOI:** 10.3389/fchem.2021.819165

**Published:** 2022-02-03

**Authors:** Zongyang Lv, Kristin E. Cano, Lijia Jia, Marcin Drag, Tony T. Huang, Shaun K. Olsen

**Affiliations:** ^1^ Department of Biochemistry and Structural Biology, University of Texas Health Science Center at San Antonio, San Antonio, TX, United States; ^2^ Department of Chemical Biology and Bioimaging, Wroclaw University of Science and Technology, Wroclaw, Poland; ^3^ Department of Biochemistry and Molecular Pharmacology, New York University School of Medicine, New York, NY, United States

**Keywords:** SARS-CoV-2, COVID-19, PLpro, Mpro, 3CLpro, papain-like protease, main protease, protease inhibitors

## Abstract

The emergence of severe acute respiratory syndrome (SARS-CoV-2) in 2019 marked the third occurrence of a highly pathogenic coronavirus in the human population since 2003. As the death toll surpasses 5 million globally and economic losses continue, designing drugs that could curtail infection and disease progression is critical. In the US, three highly effective Food and Drug Administration (FDA)–authorized vaccines are currently available, and Remdesivir is approved for the treatment of hospitalized patients. However, moderate vaccination rates and the sustained evolution of new viral variants necessitate the ongoing search for new antivirals. Several viral proteins have been prioritized as SARS-CoV-2 antiviral drug targets, among them the papain-like protease (PLpro) and the main protease (Mpro). Inhibition of these proteases would target viral replication, viral maturation, and suppression of host innate immune responses. Knowledge of inhibitors and assays for viruses were quickly adopted for SARS-CoV-2 protease research. Potential candidates have been identified to show inhibitory effects against PLpro and Mpro, both in biochemical assays and viral replication in cells. These results encourage further optimizations to improve prophylactic and therapeutic efficacy. In this review, we examine the latest developments of potential small-molecule inhibitors and peptide inhibitors for PLpro and Mpro, and how structural biology greatly facilitates this process.

## Introduction

In the past 2 decades, humans have experienced three major coronavirus outbreaks: severe acute respiratory syndrome (SARS) in 2003, Middle East respiratory syndrome (MERS) in 2012–2013 and, currently, coronavirus disease 2019 (COVID-19) since 2019. Since the first case of COVID-19 was reported in December 2019 in Wuhan, China, this disease has rapidly spread in China and around the world. In early 2020, the novel coronavirus SARS-CoV-2 was identified as the causative agent, and by March 2020, WHO characterized COVID-19 as a pandemic. This outbreak has resulted in over 240 million confirmed cases and over 5 million related deaths to date. The virus has caused huge economic loss globally due to mandatory lockdowns and quarantines.

Two major efforts from the drug discovery industry battling COVID-19 focused on developing vaccines to prevent infection and drugs to treat patients. Currently, there are three vaccines that are being administrated in the Unites States: Johnson and Johnson’s Janssen, Pfizer-BioNTech, and Moderna. The vaccines were shown to be effective in preventing infection and alleviating symptoms. However, a significant number of people remain unvaccinated. At the time of preparation of this manuscript, new cases and new variants are still emerging.

Several treatments like fever treatment, oxygen supplementation, and mechanical ventilation are used as supportive care, but a SARS-CoV-2–specific antiviral has been the focus of scientists worldwide. Activity assays, drug screening, computational analysis, and structure determination techniques have all been well developed since 2003. Drug development for COVID-19 had built upon knowledge and experience from SARS research and quickly generated exciting prospects, which will be discussed extensively below (Ho, 2003; Lapinsky and Hawryluck, 2003).

Currently, there are over 6,500 records of clinical trials on the official website (clinicaltrials.gov). However, drugs that are approved to treat COVID-19 are scarce. Veklury (Remdesivir) is a Food and Drug Administration (FDA)–approved antiviral drug that interferes with the activity of RNA-dependent RNA polymerase and is approved for use in adults and pediatric patients [12 years of age and older and weighing at least 40 kg (about 88 pounds)] for the treatment of COVID-19 requiring hospitalization (Warren et al., 2016; Siegel et al., 2017; Gupta et al., 2019; Li et al., 2020). The FDA has issued Emergency Use Authorization (EUA) for several monoclonal antibody treatments for COVID-19 for the treatment of mild or moderate COVID-19 in adults and pediatric patients (ages 12 and up) (Baum et al., 2020). In addition, now, Pfizer has announced an oral therapeutic called Paxlovid that inhibits the activity of SARS-CoV-2 main protease (Mpro) and can reduce the risk of hospitalization or death by 89% (Owen et al., 2021; Pfizer 2021). Merck has also recently announced an oral therapeutic called Molnupiravir that interferes with RNA-dependent RNA polymerase and reduces the risk of hospitalization or death by approximately 50% (Sheahan et al., 2020). Merck and Pfizer are pursuing EUA, and if granted, Paxlovid and Molnupiravir would be the first orally administered COVID-19 antiviral treatments with game changing potential in the battle against the pandemic.

Despite these many advances, the search for COVID-19–specific treatments is far from over. New SARS-CoV-2 virus strains are emerging, and some showed an increase in transmissibility and severity in infections. Development of new drugs targeting different components of the virus can potentially override the risk of new mutations. Structure-guided drug discovery has been a useful method for many viruses. Ongoing efforts to identify antivirals for SARS-CoV-2 have focused on three NSPs (nonstructural proteins): nsp3 papain-like protease (PLpro), nsp5 Mpro, and nsp12 RNA-dependent RNA polymerase. From here, we are going to look at the function and structure of the two SARS-CoV-2 proteases essential for viral replication (PLpro and Mpro) and how structural biology facilitates the development of inhibitors targeting these two proteases.

## Papain-Like Protease and Main Protease Are Two Important Proteases for SARS-CoV-2

**FIGURE 1:**
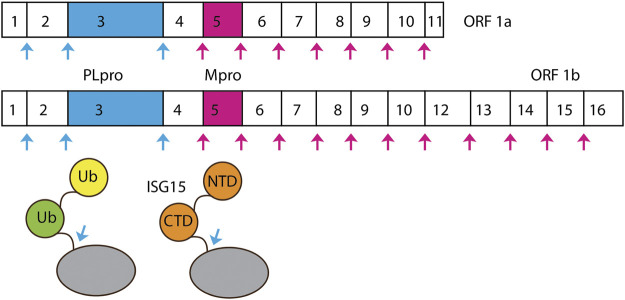
Cleavage sites of PLpro and Mpro. SARS-CoV-2 ORF1a is processed by PLpro and Mpro into Nsp1-Nsp11.

SARS-CoV-2 belongs to the clade B of genus betacoronavirus. The viral genome is made up of a single-stranded positive-sense RNA of about 29.8–29.9 kbp in size. At 5’ of SARS-CoV-2 genome, there are two overlapping ORFs: ORF1a and ORF1b. ORF1b utilizes a programmed −1 ribosomal frameshift that allows translation of nsp11-nsp16 after the stop codon of ORF1a (Figure 1) (Kelly et al., 2020; Giri et al., 2021). Other ORFs encode four conserved structural proteins—spike (S), envelope (E), membrane (M), and nucleocapsid (N)—and six accessory proteins (Kim et al., 2020). ORF1a and ORF1b encode polyprotein 1a and 1b (pp1a and pp1b), which are cleaved into 16 NSPs by protease activity of two cysteine proteases: PLpro and Mpro.

PLpro specifically identifies and cleaves peptide bonds between nsp1 and nsp2 (LNGG↓AYTR), nsp2 and nsp3 (LKGG↓APTK), and nsp3 and nsp4 (LKGG↓KIVN), liberating three proteins: nsp1, nsp2, and nsp3 (Figure 1) (Harcourt et al., 2004). In SARS-CoV-2, nsp3 contains 1,945 residues with a mass of ∼212 kDa. PLpro is a domain of nsp3—a large multi-domain protein (amino acid residues 746–1,060) that is an essential component of the replication and transcription complex (RTC) (Lei et al., 2018). The enzyme is located in nsp3 between the SARS unique domain and a nucleic acid-binding domain. It is highly conserved and found in all coronaviruses (Lei et al., 2018). When two copies are present in MERS, a single PLpro was found in SARS-CoV-1 and SARS-CoV-2 (Woo et al., 2010; Mielech et al., 2014).

In addition to its ability to hydrolyze the peptide bonds linking nsp1/nsp2, nsp2/nsp3, and nsp3/nsp4, PLpro also cleaves ubiquitin (Ub) and ISG15 [interferon (IFN)–stimulated gene 15] substrates (Figure 1) (Ratia et al., 2014; Li et al., 2016). Ub is a small regulatory protein found in most eukaryotic organisms (Komander and Rape 2012). It affects most eukaryotic cellular pathways by covalently modifying an amino group on substrates by a cascade of three enzymes: E1, E2, and E3 (Komander and Rape, 2012; Lv et al., 2017; Yuan et al., 2017; Lv et al., 2018). Ub can also serve as a substrate of ubiquitination modification on one of its amino groups and, most importantly, on the side chains of K48 and K63 forming K48-linked and K63-linked poly-Ub chains. These chains interact with different Ub binding domains and lead to protein degradation and various cellular signaling events, including innate immunity (Komander and Rape 2012). K63-linked poly-Ub was shown to activate the TAK1 kinase complex, which, in turn, phosphorylates and activates IKK (Deng et al., 2000; Wang et al., 2001). IKK phosphorylates NF-κB inhibitory proteins IκB (Karin 1999). Phosphorylated IκB is ubiquitinated by SCF complex, forming K48-linked poly-Ub chains, which is the signal for proteasome degradation. Freed NF-κB translocates into nucleus and activates transcription of a plethora of genes (Hayden and Ghosh, 2008).

ISG15 is a ubiquitin-like modifier. It is conjugated to substrate with an enzyme cascade similar to Ub (Perng and Lenschow, 2018). ISG15 is induced by type I IFN, and ISG15 can directly inhibit viral replication and modulate host immunity (Perng and Lenschow, 2018). The protease activity of SARS-CoV-2 PLpro toward K48-linked poly-Ub chains and ISG15 is important in restricting innate immunity (Perng and Lenschow, 2018; Klemm et al., 2020). With the presence of the protease activity of SARS-CoV-2 PLpro, there is a decrease in ISGylation of IFN regulatory factor 3, and decreases in phosphorylation of TBK1, which is an activation event of the NF-κB pathway (Shin et al., 2020).

Mpro is the protein encoded from nsp5. Mpro cleaves two large overlapping polyproteins pp1a and pp1ab at 11 conserved sites, including its own N-terminal and C-terminal autoprocessing sites. SARS-CoV-1 Mpro and SARS-CoV-2 Mpro exhibit highly overlapping substrate specificities (Rut et al., 2021). The enzyme has a recognition sequence of Leu-Gln↓(Ser, Ala, Gly), where ↓ marks the cleavage site (Figure 1) (Anand et al., 2003; Hilgenfeld, 2014). It is responsible for the cleavage of pp1a/1ab to produce the mature of nsp4–16. This protease is called the Mpro because it plays a major role in processing replicase polyproteins and thus facilitates viral gene expression and replication.

## SARS-CoV-2 Papain-Like Protease Structure

PLpro is a cysteine protease with rich cysteine content; in addition to catalytic C111, there are 10 other cysteines, of which four coordinate a structural zinc atom. Mutation of the cysteines coordinating zinc causes loss of activity (Barretto et al., 2005). A high concentration of reducing reagent is usually applied to keep the protein in the active state (Rut et al., 2020a); otherwise, oxidation of the catalytic cysteine is observed (Lin et al., 2018). Wild-type (WT) PLpro was also reported to have a poor crystallization property (Osipiuk et al., 2021a).

**FIGURE 2:**
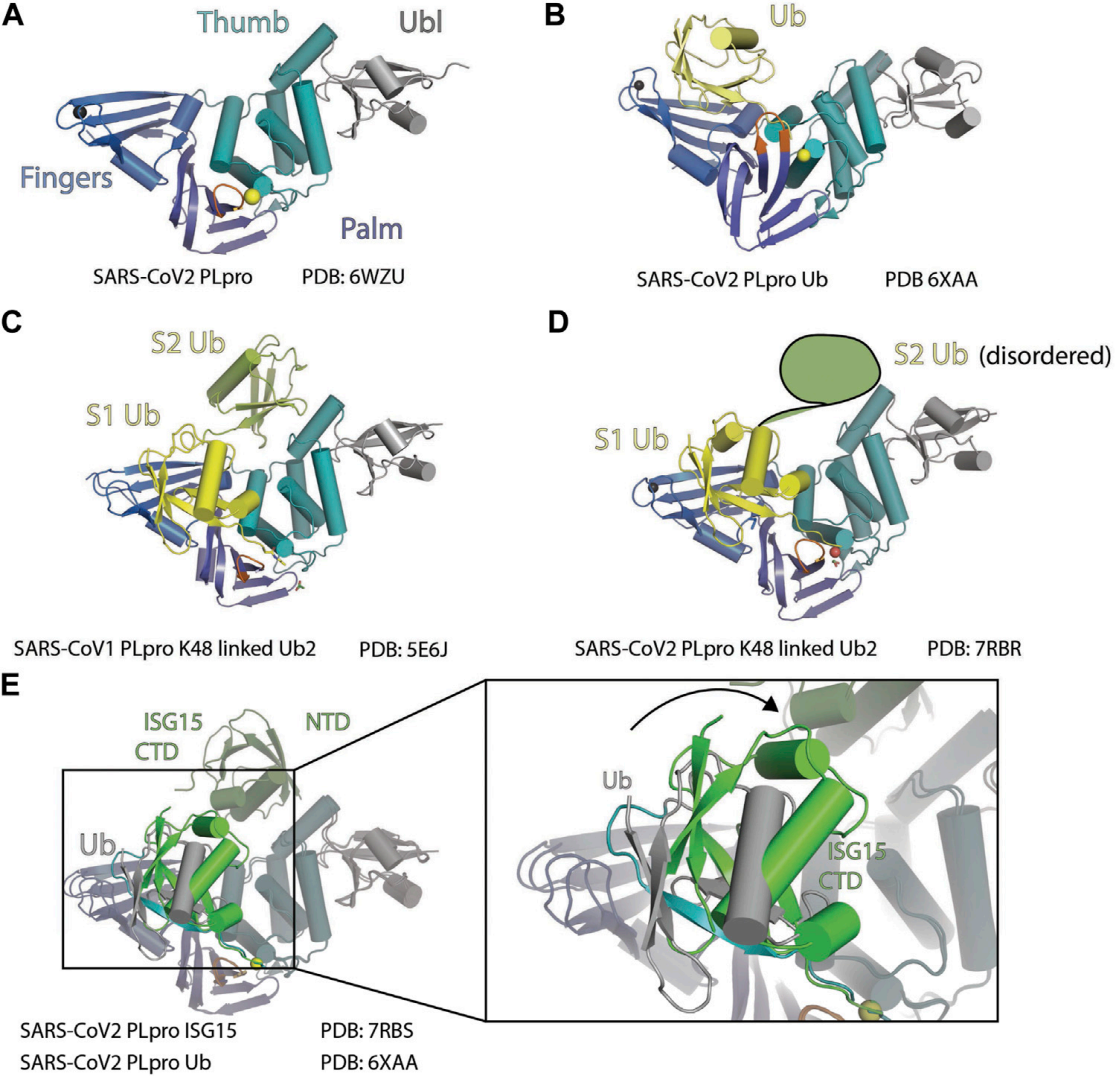
Structure of PLpro with Ubl. **(A)** Crystal structure of SARS-CoV-2 PLpro is shown as cartoon to demonstrate its four domains (PDB: 6WZU): Ubiquitin-like domain Ubl (gray), Thumb domain (teal), Palm domain (slate), and Finger domain (marine). BL loop is colored orange. Sulfur atom of C111 and Zinc ion are shown as spheres. **(B)** Crystal structure of SARS-CoV-2 PLpro in complex with ubiquitin is shown as cartoon (PDB 6XAA). PLpro is colored as in panel A and ubiquitin is colored yellow. **(C)** Crystal structure of SARS-CoV-1 PLpro in complex with K48-linked diUb is shown as cartoon (PDB 5E6J). S1 (proximal) Ub is colored yellow, and S2 (distal) Ub is colored lime. **(D)** Crystal structure of SARS-CoV-2 PLpro in complex with K48-linked diUb is shown as cartoon (PDB: 7RBR). S1 Ub is colored yellow. S2 (distal) Ub is disordered, and its position is outlined. **(E)** Crystal structure of SARS-CoV-2 with Ub (PDB: 6XAA) is superimposed with the crystal structure of SARS-CoV-2 with ISG15 (PDB: 7RBS). The rotation of ISG15 CTD compared to Ub at S1 site is highlighted.

SARS-CoV-2 PLpro lacks the N-terminal M1 residue compared to SARS-CoV-1 PLpro, which results in being smaller by one residue (Patchett et al., 2021), but maintains 83% sequence identity to SARS-CoV-1 PLpro. Several structures of apo SARS-CoV-2 PLpro have been reported, including WT structures (PDB: 6WZU, 7JRN, and 7NFV) (Osipiuk et al., 2021a) and C111S mutant structures (PDB: 7CJD, 6WRH, 6XG3, 7D47, 7M1Y, and 7K7K) (Osipiuk et al., 2021a; Gao et al., 2021; Zhao et al., 2021). The overall structure SARS-CoV-2 PLpro is similar to PLpro from SARS-CoV-1. It has a Ubl domain whose function is unknown, and a catalytic unit with a right-hand scaffold that is comprised of three domains Finger, Palm, and Thumb (Figure 2A). The Thumb–Palm–Fingers catalytic unit and the conserved catalytic triad resemble the structure of Ub-specific proteases (USPs), although with low sequence identity, whereas the Ubl domain is not present in USPs (Mielech et al., 2014; Hilgenfeld, 2014). Thumb domain is comprised of six *α*-helices and a small *ß*-hairpin. The Finger subdomain is made of six *ß*-strands and two *α*-helices and includes a zinc-binding site formed by four cysteine residues (C189, C192, C224, and C226). Zinc binding is essential for structural integrity and protease activity (Barretto et al., 2005). The Palm domain is comprised of six *ß*-strands. The catalytic residues C111, H272, and D286 are located at the interface between the Thumb and Palm domains. Most variations in the structures are at Finger domain and G266–G271 loop (also named BL2 loop or BL loop) containing Y268 and Q269 (Figure 2A) (Rut et al., 2020a; Shin et al., 2020; Smith et al., 2020; Ma et al., 2021a; Osipiuk et al., 2021a; Fu et al., 2021; Gao et al., 2021; Shan et al., 2021; Shen et al., 2021). This loop adopts different conformations in structures of the PLpro in different states: apo, substrate bound, and different inhibitor bound.

## SARS2-CoV-2 Papain-Like Protease Interacts With Ubiquitin and Ubiquitin-Like Modifier ISG15 at S1 and S2 Sites

Like the three Ub binding sites (S1’, S1, and S2) arrangement observed in the USP family of deubiquitinases (DUBs), S1–S2 sites of SARS-CoV-1 PLpro have been well characterized to interact with Ub and ISG15. Although the K48-linked diUb and two tandem Ubl domains of a single ISG15 sit on the S1 and S2 sites share the same arrangement, there are significant differences in how the Ubl domains sit on the enzyme.

At S1 of SARS-CoV-2 PLpro, both hydrophobic and hydrophilic interactions are involved in the contact with Ub (Figure 2B) (PDB: 6WZU). L199 and Y207 from Finger domain of PLpro make hydrophobic contacts with Ub globular domain. E167 from Thumb domain forms salt bridge with R42, T225, and R232 from Finger domain, M208 from Palm domain, and R166 from Thumb domain are involved in hydrogen bonds (H bonds) contacts with Ub (Chou et al., 2014; Ratia et al., 2014). E168A and E168R mutants from SARS-CoV-1, which have equivalent position of E167 of SARS-CoV-2, do not affect peptide substrate cleavage and greatly decreased DUB activity in SARS-CoV-1 PLpro (Chou et al., 2014). This validates the S1 site interaction between PLpro and Ub yet indicates that the ORF1a peptide cleavage utilizes an alternative binding mechanism independent of S1.

The structure of a K48-linked di-Ub with SARS-CoV-1 PLpro complex reveals an extended di-Ub binding and conformation across both S1 and S2 sites, rather than sitting across the S1–S1’ position; this makes SARS PLpro specific for cleavage of K48-linked polyubiquitin chains (Figure 2C) (Békés et al., 2016). This is consistent with the observation that di-Ub K48-linked chain by itself is a competing substrate and is resistant to cleavage by PLpro (Ratia et al., 2014). The position of S1 Ub in this structure is similar to the mono-Ub SARS PLpro structures. At the S2 site, Ub contacts the residues 62–74 from the *α*-helix following Ubl domain with the hydrophobic I44 patch. As a result, the K48-linked di-Ub bound to SARS PLpro is stabilized in an extended conformation that is different from prior structures of K48-linked poly-Ub chains (Cook et al., 1992).

On the basis of the high sequence similarity between PLpro from SARS-CoV-1 and SARS-CoV-2, it was expected that the two proteases process K48-linked poly-Ub chains and ISG15 modification similarly. However, several research groups independently reported PLpro from the two viruses have differences in their activity toward K48-linked poly-Ub chains (Klemm et al., 2020; Shin et al., 2020; Rut et al., 2020a; Patchett et al., 2021; Osipiuk et al., 2021b). Interestingly, Ub interacts with SARS-CoV-1 and SARS-CoV-2 PLpro at S2 and S1 sites very similarly besides minor differences caused by sequence variation between SARS-CoV-1 and SARS-CoV-2 PLpro (Shin et al., 2020; Rut et al., 2020a; Patchett et al., 2021). A recent structure of SARS-CoV-2 PLpro with Lys48-linked di-Ub shows a highly similar structure (Figure 2D) (Osipiuk et al., 2021b). A S2 site mutation (F69S/E70K/H73G) was shown to greatly reduce Ub chain cleavage activity by SARS PLpro (Patchett et al., 2021).

At S1, SARS-CoV-2 has T225 compared to V226 in SARS-CoV-1, and SARS-CoV-2 has K232 compared to Q233 in SARS-CoV-1 (Patchett et al., 2021). SARS-CoV-1 PLpro amide nitrogen from the side chain of Q233 forms an H bond with the backbone carboxylate of A46, and in SARS-CoV-2, PLpro side chain amine group of K232 also forms H bond with backbone carboxylate of A46. SARS-CoV-1 PLpro V226 forms hydrophobic contacts with the backbone of E64 and S65, whereas SARS-CoV-2 PLpro T225 forms hydrophobic contact with the side chain of Q62 and the main chain of E64 and S65. Swapping residue between SARS-CoV-1 and SARS-CoV-2 PLpro changes the features of these two proteases, proving that these minor differences in contacts are important for accounting the difference in activities toward K48-linked Ub chain between PLpro from SARS-CoV-2 and SARS-CoV-1 (Patchett et al., 2021). Shin et al. found a T75L mutant partially recovered cleavage by SARS-CoV-2 PLpro toward K48-linked poly-Ub chains (Shin et al., 2020). Our research shows that the S2 mutant T75L/D179E only partially recovers SARS-CoV-2 PLpro activity toward K48-linked poly-Ub cleavage, whereas the S1 mutant T225V/K232Q significantly improves SARS-CoV-2 PLpro cleavage of K48-linked poly-Ub chains. The corresponding swapped mutant in SARS-CoV-1 PLpro (V226T/Q233K) has reduced activity (Patchett et al., 2021). These results indicated that the differences in the primary sequence at both S1 and S2 both contributed to the difference in the activity of SARS-CoV-1 and SARS-CoV-2 PLpro toward K48-linked Ub, and variation in S1 may take a dominant role. In the recent structure of SARS-CoV-2 PLpro with K48-diUb, only weak electron density is observed for the distal domain (Osipiuk et al., 2021b). This also supports the concept of the S1 site as a major driver for Ub chain substrate recruitment.

ISG15 has two tandem Ub-like folds: NTD and CTD. ISG15 binds to PLpro in a S1–S2 arrangement similar to K48-linked diUb, with the CTD occupying S1 and NTD occupying S2, yet there are apparent differences. At S1 site, ISG15 CTD has different binding modes with PLpro compared to Ub (5TL6 (Daczkowski et al., 2017), 6XA9 (Klemm et al., 2020), and 6YVA (Shin et al., 2020). When crystalized with SARS-CoV-2 PLpro, ISG15 CTD shows a ∼40° rotation compared to S1 Ub (Figure 2E) (Klemm et al., 2020; Shin et al., 2020). As a result, ISG15 CTD loses contact with the Finger domain and gains contact with the Thumb domain, where a new set of contacts is formed including PLpro S170, Y171, and Q174, contacting G126, P128, and E130 from ISG15 (Klemm et al., 2020; Shin et al., 2020; Patchett et al., 2021). MERS PLpro, SARS-CoV-1, and SARS-CoV-2 PLpro share the same binding mode to the ISG15 CTD (Daczkowski et al., 2017; Clasman et al., 2020; Shin et al., 2020). In SARS-COV-1, based on different binding modes of Ub and ISG15, PLpro N156E resulted in selective decrease of activity in ISG15 cleavage assays, with minor impact on Ub cleavage (Békés et al., 2016). We recently found that S170A/Y171A/Q174A triple mutant is active on mono-Ub but deficient in ISG15-VS labeling (Patchett et al., 2021).

At S2, the structure of SARS-CoV-2 PLpro engages ISG15 NTD. In comparison to the free ISG15 structure, ISG15 NTD rotates about 90°, similar to the conformation when bound to MERS PLpro (Daczkowski et al., 2017; Shin et al., 2020). Comparison of binding modes at the S2 site of ISG15 and K48-linked diUb shows both Ubl domains sit on a hydrophobic site around F69 for SARS-CoV-2 and F70 for SARS-CoV. Distal Ub uses its I44 patch to interact with F70 and flanked by L8 and H68, whereas ISG15 uses M23 and an aliphatic part of E27 side chain to interact with F70. As a result, the globular domain of ISG15 NTD and distal Ub are rotated relative to each other (Békés et al., 2016; Shin et al., 2020).

### P1–P4 Sites

**FIGURE 3:**
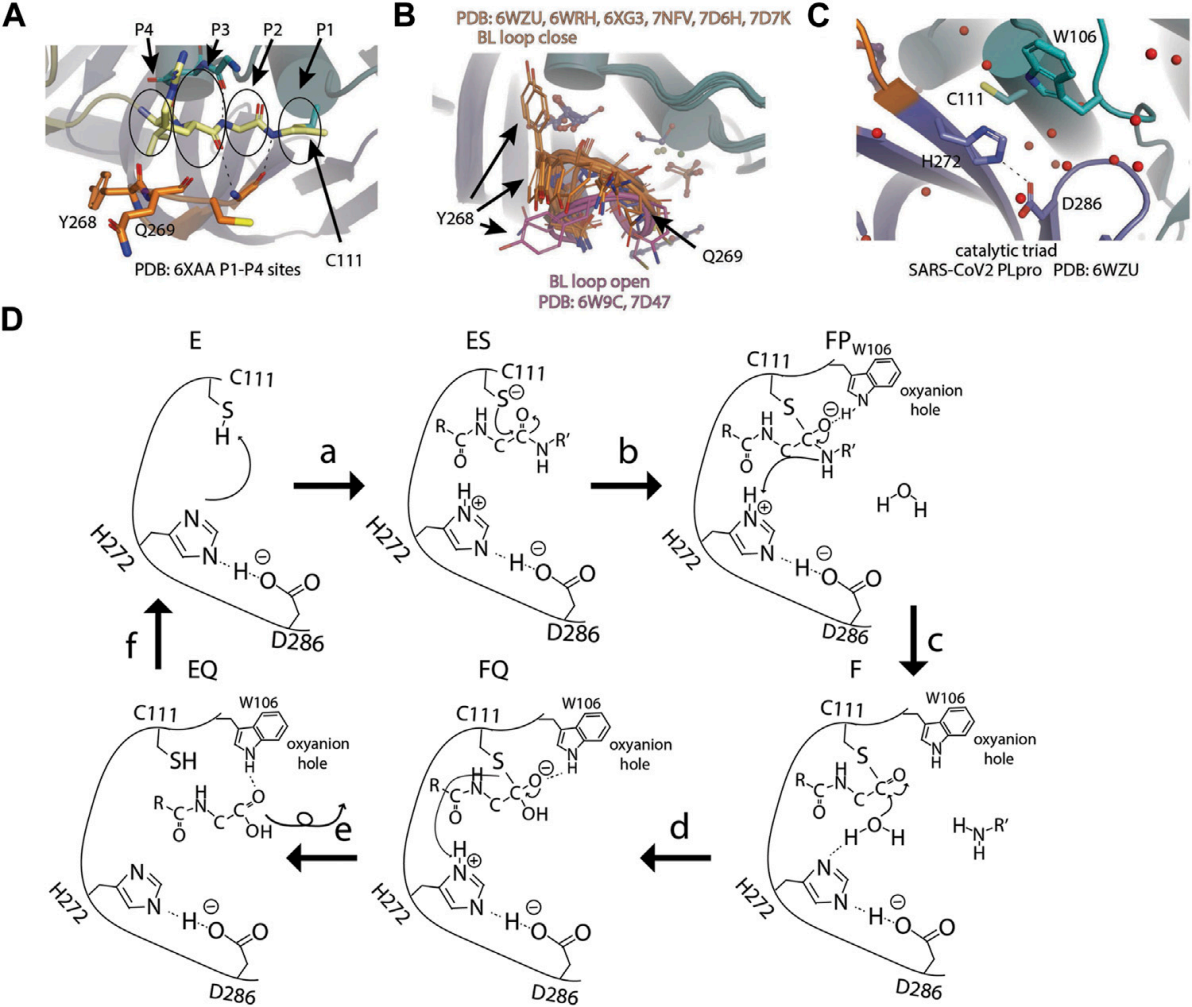
Active site and catalysis. **(A)** Close-up view of catalytic pocket PLpro with Ub bound. The last four residues of Ub occupy P1–P4 sites of the substrate binding pocket: P1 by G76, P2 by G75, P3 by R74, and P4 by L73. P1–P4 sites are highlighted by circles. **(B)** Apo structures of SARS-CoV-2 PLpro are superimposed and shown as cartoon (PDB open: 6W9C and 7D47; close: 6WZU, 6WRH, 6XG3, 7NFV, 7D6H, and 7D7K). Side chains of residue Y268 and Q269 are shown as thin sticks. BL loops in open conformation are colored pink and closed. Conformations are colored orange. **(C)** Close-up view of catalytic triad of SARS-CoV-2 PLpro (PDB 6WZU). **(D)** Schematic drawing of catalytic cycle of SARS-CoV-2 PLpro. In unliganded “E” state, the imidazole group of H272 attacks C111 thiol group to lower its pKa. In the “ES” state, when substrate enters the active site, thiolate attacks the carbon atom of amide bond and forms the first tetrahedral intermediate (“FP” state). The negative charge is transferred to amide oxygen and is stabilized by the oxyanion hole. The amine product is release upon breakage of peptide bond (“F” state). A water molecule attacks the carbonyl and forms the second tetrahedral intermediate (“FQ” state). Lastly, the elimination of cysteine from the intermediate frees the N-terminus of the substrate (“EQ” state) and the enzyme is restored to the “E” state.

Close to the catalytic site, four C-terminal residues (73–76) of Ub are bound to the narrow active site channel of PLpro (Chou et al., 2014). The positions occupied by the last four residues of Ub were named P4 (L73), P3 (R74), P2 (G75), and P1 (G76) sites. Amino acid residues around the P4–P1 sites are conserved between SARS-CoV-1and SARS-CoV-2, including the conserved catalytic triad of PLpro. The substrate binding channel is very narrow at the P1 and P2 sites, consistent with the high specificity of glycine residues at these two sites (Figure 3A) (Rut et al., 2020a). The P1 and P2 sites have polar interactions with substrate, including H bond with G271 and G163, and van der Waals contacts to L163 and Y164. The substrate binding channel becomes solvent exposed at P3 site and wide at the P4 site to accommodate larger side chains of leucine and arginine. Importantly, the loop *β*11–12 strand or BL loop forms the boundary of P3–P4 sites (Figure 3A) (Hu et al., 2005). The BL loop is highly dynamic among apo structures, and it adopts different conformations, including its movement in both backbone and side chains. In PDB accessions 7D47 and 6W9C, BL loops are in an open conformation, whereas in PDB accessions 6WZU, 6WRH, 6XG3, 7NFV, 7D6H, and 7D7K, the BL loops are closed (Figure 3B). Adding to the backbone movement, the side chains of two residues Y268 and Q269 adopt various rotamers. Upon binding of substrate, BL loop closes and locks substrate in position for catalysis. G271 forms H bond with G76 from Ub. Y268 and Q269 are involved in van der Waals contact with L71, R72, L73, and R74 from Ub. The plasticity of Y269 from SARS PLpro was exploited for drug discovery targeting Baez-Santos et al. (Báez-Santos et al., 2014a; Báez-Santos et al., 2015). New inhibitors targeting SARS-CoV-2 also take advantage of plasticity in this region involving corresponding residue Y268, which will be discussed in the following sections.

### Catalytic Triad

Next to the P1 site, SARS-CoV-2 PLpro has a canonical cysteine protease catalytic triad comprising C111, H272, and D286) (Báez-Santos et al., 2015; Rut et al., 2020a; Shin et al., 2020; Osipiuk et al., 2021a) (Figure 3C) D286 forms an H bond with the side chain of H272, therefore, restricting its rotation. This action aligns H272, so its side chain faces C111 for catalysis. In the first step, C111 is deprotonated by the basic side chain of H272 to increase its reactivity. Then the amide bond of substrate is nucleophilic attacked by the deprotonated C111. This results in the formation of tetrahedral intermediate and subsequent breakage of amide bond. C111 forms a thioester intermediate with the carboxyl-terminus (C-terminus) of the substrate. The carboxyl oxygen under attack now has a negative charge and is stabilized by the oxyanion hole including W106. H272 protonates the amine and restores its deprotonated form. The thioester bond is subsequently hydrolyzed, releasing the carboxylic acid substrate fragment, and the enzyme is restored (Figure 3D).

## Inhibitors Against SARS-CoV-2 Papain-Like Protease

### GRL0617 and Its Analogs Inhibit SARS-CoV-1 Papain-Like Protease

In 2008, Ratia et al. screened a structurally diverse library of 50,080 compounds for inhibitors of PLpro with RLRGG-AMC (7-amido-4-methylcoumarin) fluorescent substrate. Authors included 5 mM DTT in the assay to prevent electrophiles from non-specifically binding the catalytic cysteine (Ratia et al., 2008). This screening campaign found compound 7724772 that inhibited PLpro with an IC_50_ (half-maximal inhibitory concentration) value of 20.1 ± 1.1 μM (Table 1). A series of derivatives were synthesized and tested for potency. Refinement by the addition of a naphthyl group and an amino group to the ortho-methyl benzene ring resulted in the more potent compound GRL0617 (Table 1). It has an IC_50_ = 0.6 ± 0.1 μM toward SARS-CoV-1 PLpro. Compound 6 has one more Ac group than GRL0617, and it has decent potency with IC_50_ = 2.6 μM and EC_50_ (half-maximal effective concentration) = 13.1 μM (Table 1) (Ratia et al., 2008). Both GRL0617 and compound 6 inhibited SARS-CoV-1viral replication in Vero E6 cells with an EC_50_ value of 14.5 and 13.1 μM, respectively. It is also encouraging that they had no associated cytotoxicity (Ratia et al., 2008).

**TABLE 1:** GRL0617-like inhibitors I.

Compound Name	Chemical Structure	IC50	EC50	References
7724772	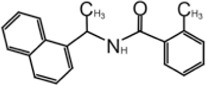	20.1 ± 1.1 μM	—	Ratia et al. (2008)
GRL0617	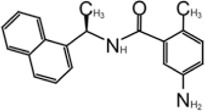	∼2 μM	∼20 μM	Barretto et al. (2005); Beigel et al. (2020); Fu et al. (2020); Hoffman et al. (2020); Ahmad et al. (2021); Amporndanai et al. (2021); Fu et al. (2021)
Compound 6	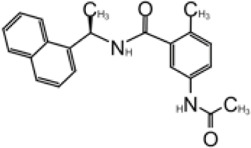	11 ± 3 μM	—	Ahmad et al. (2021)
Compound 2	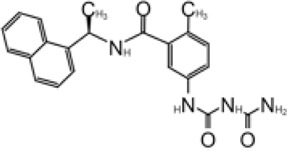	5.1 ± 0.7 μM	Failed	Osipiuk et al. (2021a)
Compound 3	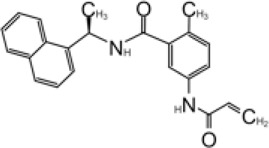	6.4 ± 0.6 μM	Failed	
Compound 5	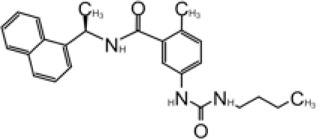	16.8 ± 2.9 μM	2.5 μM	
ZN2-184	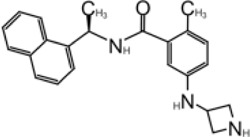	1.01 ± 0.15 μM	—	Shen et al. (2021)
ZN-3–80	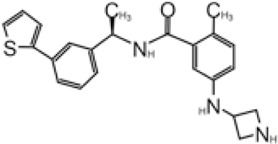	0.59 ± 0.04 μM	—	
XR8-23	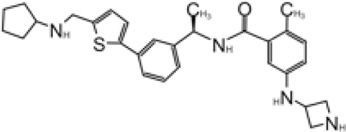	0.39 ± 0.05 μM	2.8 ± 0.4 μM	
XR8-24	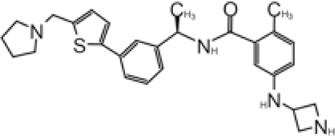	0.56 ± 0.03 μM	2.5 ± 0.3 μM	
XR8-89	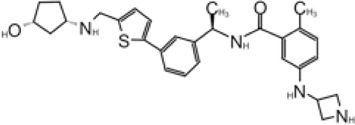	0.113 ± 0.004 μM	11.3 ± 1.6 μM	

**FIGURE 4:**
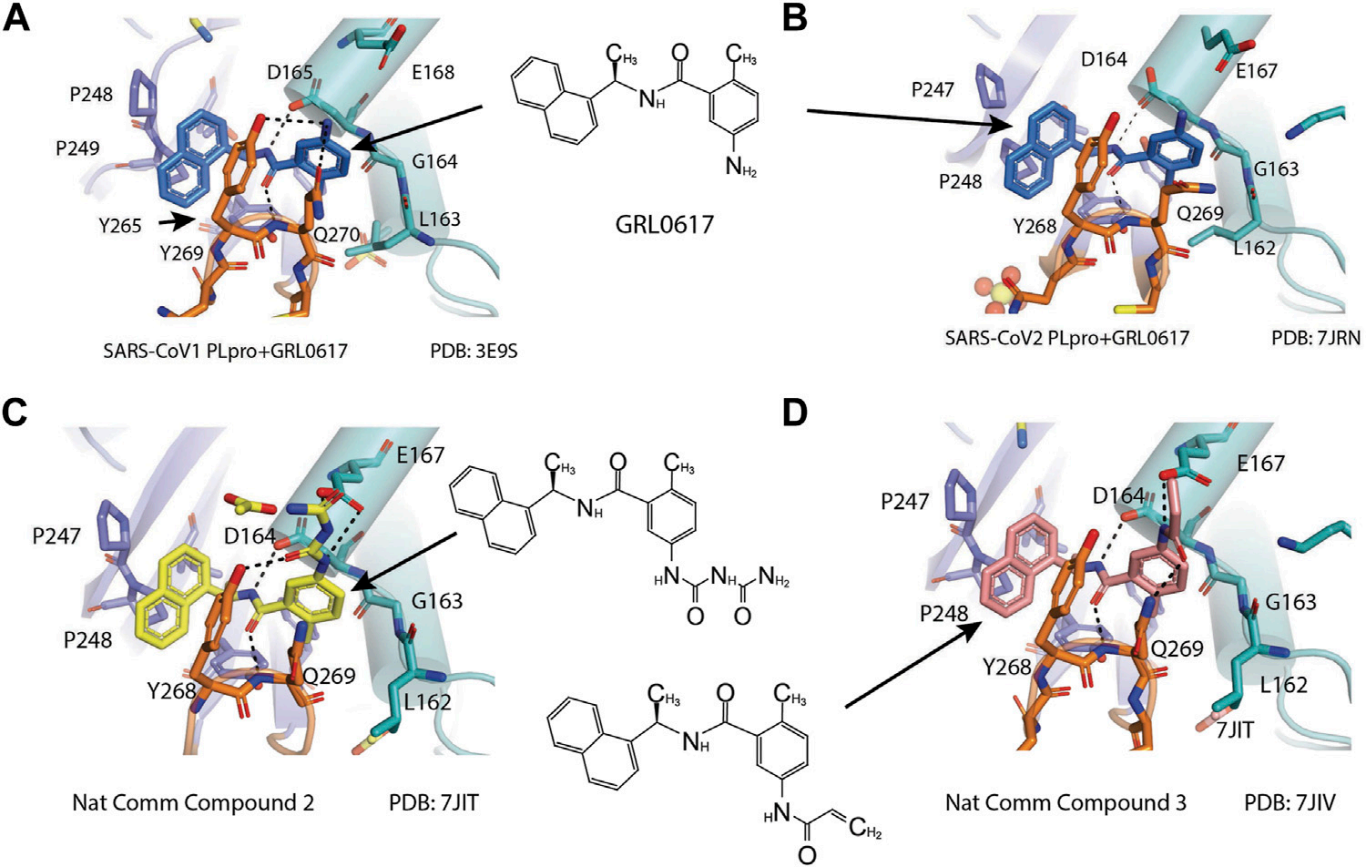
Close-up view of interaction between SARS-CoV-1 PLpro and inhibitors in crystal structures. PLpro is shown as cartoon with sticks representation shown for residues involving contact with inhibitors. Inhibitors are shown as sticks. Hydrogen bonds are labeled with dashed lines. **(A)** SARS-CoV-1 PLpro with GRL0617 (PDB: 3E9S). **(B)** SARS-CoV-2 PLpro with GRL0617 (PDB: 7JRN). **(C)** SARS-CoV-2 PLpro with compound 2 (PDB: 7JIT). **(D)** SARS-CoV-2 PLpro with compound 3 (PDB: 7JIV).

X-ray structure of the SARS-CoV-1 PLpro-GRL0617 complex was solved at a resolution of 2.5 Å (Figure 4A). The structure shows that the GRL0617 binds at P3–P4 position, in proximity but not within the catalytic site. The interaction between GRL0617 and PLpro is stabilized through H bonds and hydrophobic interactions. The 1-naphthyl group forms hydrophobic interactions with the aromatic rings of Y265 and Y269. P248 and P249 residues line the substrate binding pocket, and they are known to accommodate the leucine residue at the P4 position of PLpro substrates (Figure 4A) (Ratia et al., 2006). The di-substituted benzene ring occupies the putative P3 position and stacks against the aliphatic portions of G164, D165, and Q270. The ortho-methyl group is lined by the side chains of Y265, Y274, and L163, and the amino group is surrounded by the side chain oxygen of Q270 and E168 and the hydroxyl of Y269 (Figure 4A) (Ratia et al., 2008). Comparison of the unbound and inhibitor-bound structures reveals a significant conformational difference in the BL loop that it moves toward GRL0617 and gains contacts with the inhibitor. Along with the movement of backbone, the side chains of Y269 and Q270 close over the inhibitor (Figure 4A) (Ratia et al., 2008). Importantly, GRL0617 was unable to inhibit HAUSP, USP18, UCH-L1, UCH-L3, and a papain-like protease (PLP2) from the human coronavirus NL63. The high specificity and low cytotoxicity make GRL0617 an ideal lead for future refinement (Ratia et al., 2008; Ghosh et al., 2009).

### GRL0617 is Also a Good Inhibitor for SARS-CoV-2 Papain-Like Protease

As the catalytic site including P1–P4 is strictly conserved between S1 and SARS-CoV-2 PLpro, Brendan et al. tested five inhibitors including 7724772 and GRL0617. It was found that GRL0617 inhibited SARS-CoV-2 PLpro IC_50_ value of 2.4 μM (Freitas et al., 2020). Inhibition of PLpro by GRL0617 was used to confirm the role of PLpro in modulating host immunity through IFN and NF-κB pathways (Shin et al., 2020). Freitas et al. also used GRL0617 directly and found it inhibited SARS-CoV-2 PLpro with IC_50_ = 2.4 μM (Freitas et al., 2020). GRL0617 is often among the best hits from high-throughput screening campaigns or used effectively as a positive control (Smith et al., 2020; Zhao et al., 2021; Shen et al., 2021; Shan et al., 2021; Fu et al., 2021). Smith et al. (2020) screened several libraries and found disulfiram and GRL0617 as the best leads (Smith et al., 2020). Fu et al. (2021) showed that GRL0617 inhibited the deISGylation activity of PLpro in a cell-based assay. The *in vitro* IC_50_ values of GRL0617 against SARS-CoV-2 PLpro were 2.1 ± 0.2 μM (Fu et al., 2021). Shen et al. used an unbiased ChemDiv library (10,000-compound SMART library subset excluding PAINS compounds) and a biased, annotated TargetMol Bioactive library (5,370 compounds) to screen for inhibitors against SARS-CoV-2 PLpro. This screen resulted in a low hit rate, identifying only CPI-169 and the positive control GRL0617 (Table 1) (Shen et al., 2021). Jerzy et al. tested GRL0617 (named compound 1 in their paper) at IC_50_ value of 2.3 μM *in vitro* (Osipiuk et al., 2021a). Shan et al. first screened 25 DUB inhibitors and only found GRL0617 (Shan et al., 2021). Authors then screened 35,360 diverse compounds, including lead-like fragments, FDA-approved drugs, and small molecules with reported biological activities and follow-up assays, and found that GRL0617 was the best hit in potency, selectivity, and molecular complexity (Shan et al., 2021). These efforts show GRL0617 is indeed a good lead for inhibition of SARS-CoV-2 PLpro.

The mechanism of inhibition by GRL0617 has been investigated, and Shin et al. showed GRL0617 is ineffective against MERS-PLpro; authors hypothesized that this could be due to the presence of threonine instead of tyrosine at this conserved position (Y268 in SARS-CoV-2 PLpro) (Shin et al., 2020). Accordingly, the mutation of Y268 to either threonine (Y269T) or glycine (Y268G) in SARS-CoV-2 PLpro strongly reduced the inhibitory effect of GRL0617 (Shin et al., 2020). It was believed that GRL0617 functions by blocking the entry of the Ub and ISG15 C-terminus toward the catalytic cleft of the protease, as it occupies the P3–P4 position (Ratia et al., 2008; Shin et al., 2020). Indeed, Fu et al. used NMR to show that ^15^N-ISG15 caused drastic peak broadening and intensity loss in the ^1^H,^15^N-HSQC NMR spectrum with PLpro, and it was recovered by titration of GRL0617, proving the concept that GRL0617 competes with ISG15 for the binding site in PLpro and blocks the binding of Ubl to PLpro (Fu et al., 2021).

Four structures of SARS-CoV-2 PLpro with GRL0617 were reported (Osipiuk et al., 2021a; Gao et al., 2021; Ma et al., 2021a; Fu et al., 2021). Compared to the apo-structure of PLpro C111S (PDB: 6WRH), consistent with previous observations in SARS-CoV-1 PLpro, there is an apparent conformational change of the BL loop that stabilizes GRL0617 binding. The structures show consistent binding mode that GRL0617 occupies the P3–P4 positions of the substrate cleft near the active site (Figure 4A). The BL loop connecting *α*3 and *α*4 forms one side of the boundary of this pocket and closes toward the inhibitor compared to apo conformation. This movement is consistent with the observation from SARS-CoV-1 PLpro structures (Ratia et al., 2008). Side chains of both Y268 and Q269 close toward GRL0617 (Figure 4A). The movement of both backbone and side chains of residues uncover hydrophobic region and form polar and hydrophobic interactions with GRL0617. Y269 wedges between substituted benzene group and 1-naphthyl group (Figure 4A). Aliphatic region of Q269 forms van der Waals contact with the benzene ring. The H bonds and hydrophobic interactions between GRL0617 and PLpro are conserved from SARS-CoV-1 to SARS-CoV-2. The 1-naphthyl group forms hydrophobic interactions with the aromatic rings of Y264 and Y268, and it is partially solvent-exposed. P247 and P248 residues set important boundaries for the substrate binding pocket (Figure 4A). The (R)-methyl group points toward Y264 and T301. The carbonyl oxygen of GRL0617 forms an H bond with the backbone nitrogen of N269 (Figure 4A). The di-substituted benzene ring occupies the putative P3 position and stacks against the aliphatic portions of G163, D164, and Q269. The ortho-methyl group is lined by the side chains of Y264, Y273, and L162, and the amino group of aniline is surrounded by the side chain oxygen of Q269 and E167 and the hydroxyl of Y268, forming H bonds with side chain of Y268 and potentially E167 (Figure 4A) (Ma et al., 2021a; Osipiuk et al., 2021a; Fu et al., 2021; Gao et al., 2021). Some minor differences are observed in the three GRL0617 bound CoV-2 PLpro structures. In PDB accessions 7CMD and 7JRN, the side chain of L162 is about 3.7 Å away from ortho–methyl group, whereas in PDB accessions 7JIR and 7CJM, L162 is an outlier that its side chain flips away from GRL0617 and has no contact with the inhibitor. Another amino acid residue that shows a difference is E167 from PDB 7JIR and 7CJM, whose side chain oxygen is ∼3.7 Å from the aniline amine group of GRL0617, likely forming a weak H bond, whereas the same side chains from the other PDBs indicate side chains of E167 flipping away from inhibitor (Ma et al., 2021a; Osipiuk et al., 2021a; Fu et al., 2021; Gao et al., 2021).

### Development of GRL0617 Derivatives

The inhibition, structure, and effectiveness of GRL0617 against SARS-CoV-2 PLpro are all in agreement with previously observations with SARS-CoV-1 PLpro. GRL0617 is a promising platform for further development, especially considering its low cytotoxicity and good potency. Some representative compounds are discussed below. Compound 6 (Table 1) was initially reported among a series of derivatives of the initial hit 7724772, along with GRL0617 (Ratia et al., 2008). It was generated by adding an acetyl group to GRL0617. For SARS-CoV-1 PLpro, Compound 6 has similar IC_50_ value with GRL0617, whereas its EC_50_ at 13.1 μM is similar to GRL0617 (Ratia et al., 2008). Freitas et al. report that compound 6 has an IC_50_ value of 5 μM (Freitas et al., 2020). More recently, Fu et al. showed that the *in vitro* IC_50_ values of compound 6 against SARS-CoV-2 PLpro were 11 ± 3 μM (Fu et al., 2021).

D164 and E167 are in proximity of the amine of four methyl aniline groups from GRL0617, and Jerzy et al. generated a series of GRL0617 derivatives to derivatize in this region (Osipiuk et al., 2021a). Crystal structures of SARS-CoV-2 PLpro with compounds 2 and 3 were achieved (Table 1) (Figures 4C,D) (PDB: 7JIT, 7JIV, and 7JIW). As expected, the inhibitors bind to the same site in the enzyme as GRL0617, located 8–10 Å away from the catalytic cysteine. Some of the newly designed inhibitors had additional contacts. For example, compound 2 has interactions with its carbamylurea moiety, forming H bonds with Glu167, Tyr268, and water-mediated H bond with K157 (Figure 4C), yet these derivatives including compound 2 had decreased potency (IC_50_ in the range of 5.1–32.8 μM) for unknown reasons. The new inhibitors were also tested in Vero E6 cells for the SARS-CoV-2 replication. Interestingly, the viral replication assay shows different comparison of potency among inhibitors with biochemical assay. Compounds 2 and 3 are good PLpro inhibitors with IC_50_ values of 5.1 and 6.4 μM, respectively, but failed in the viral replication assay. Compound 5 was the weakest inhibitor *in vitro* with IC_50_ values of 32.8 μM, but it was one of the best performers in the live viral replication assay (EC_50_ = 2.5 μM). The authors speculated that the differences in cell permeability and solubility could account for the differences (Osipiuk et al., 2021a).

**FIGURE 5:**
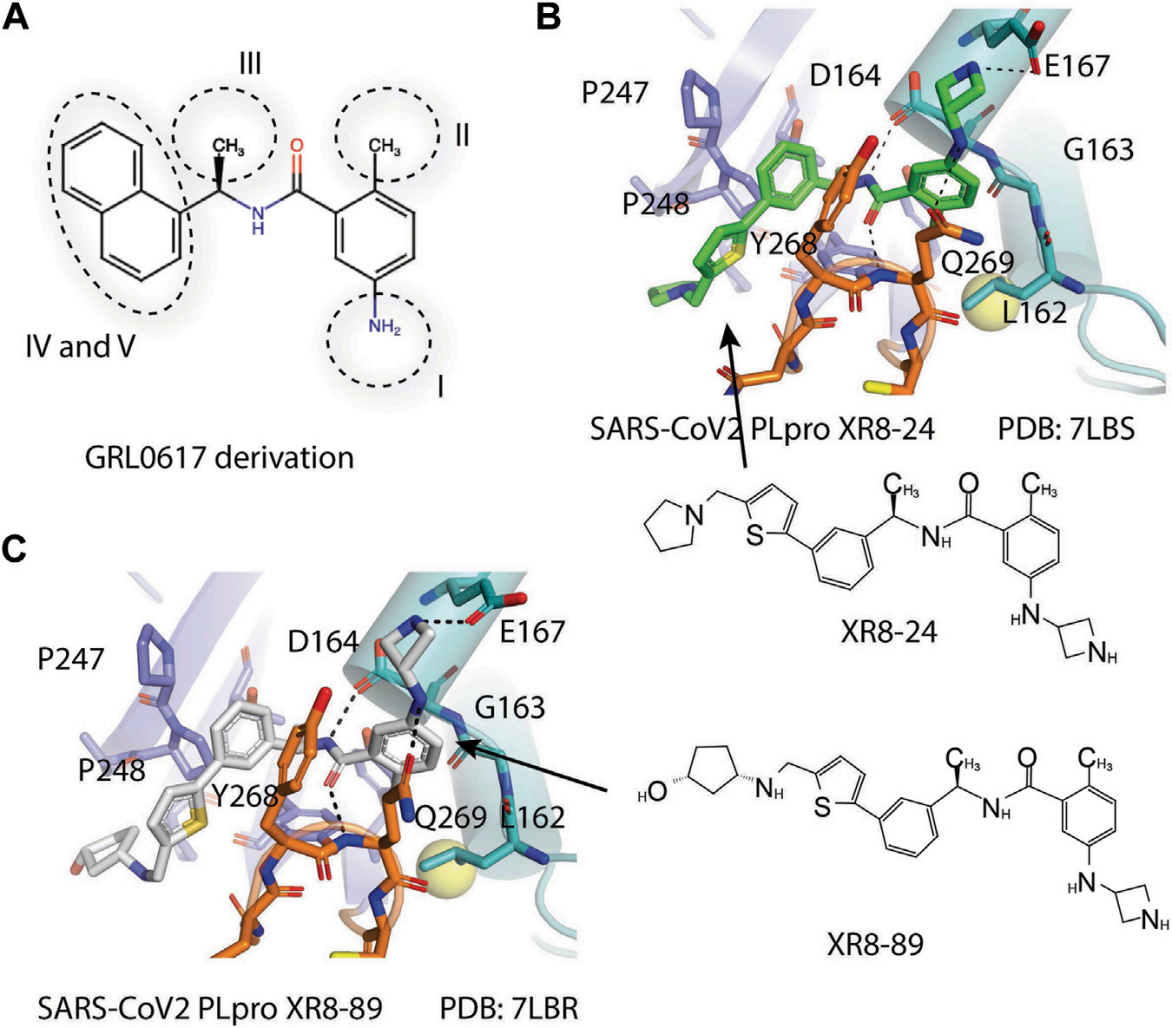
**(A)** Five regions that were derivatized for refinement of GRL0617. **(B** and **C)** Close-up view of interaction between SARS-CoV-1 PLpro and inhibitors in crystal structures. PLpro is shown as cartoon with sticks representation shown for residues involving contact with inhibitors. Inhibitors are shown as sticks. Hydrogen bonds were labeled with dashed lines. **(B)** SARS-CoV-2 PLpro with XR8-24 (PDB: 7LBS). **(C)** SARS-CoV-2 PLpro with XR8-89 (PDB: 7LBR).

Shen et al. used a more systematic approach that derivatized five regions of GRL0617 (Figure 5) (Shen et al., 2021). Region I is from the amine group of aniline; region II is from the ortho methyl group on derivatized benzene group; region III is from the R methyl group; region IV and region V are achieved by replacing the naphthalene group and further extensions (Figure 5A) (Shen et al., 2021). Refinement at region II (replacing ortho methyl group with -Cl, -Br, -CH = CH, -CF3, or -F) was not successful. Although extra room at region III suggested room for refinement (Ratia et al., 2008), replacing the (R)-methyl group with–Et, –CH_2_CH_2_OH, or–CH_2_CONHCH_3_ group decreased potency (Shen et al., 2021). The generated derivatives show lower potency than GRL0617. At region I, adding azetidine group to derivatize the amine group from aniline yielded ZN2-184 that has two-fold increase in potency (Table 1). The rational of this modification is the same as Jerzy et al., to gain contact with E167, and Shen et al. found more a favorable group at this site (Shen et al., 2021). At region IV, replacing naphthalene ring with fused heteroaryls, such as benzothiophene, indole, and carbazole with various linkages, had lower potency, likely due to spatial restraints. Replacement of naphthalene with bi-aryl groups like 2-phenylthiophene (ZN-3–80; IC_50_ = 0.59 μM) increased potency (Table 1). Taking advantage of the extra space next to the naphthalene group, which features hydrophobic residues like P248 and P299, and backbone of G266 (named BL groove), adding basic groups from phenylthiophene significantly improved potency, dropping IC_50_ to below 500 nM. XR8-89 is the best in the series and has an IC_50_ value of 0.11 μM (Table 1).

SPR assays show the extended ligands with basic side chain have significant decreased dissociation rates of XR8-89 and XR8-23 (Shen et al., 2021). To examine the binding mode of the novel PLpro inhibitors, authors obtained co-crystal structures of XR8-24, XR8-65, XR8-69, XR8-83, and XR8-89 with SARS-CoV-2 PLpro (PDB: 7LBR, 7LBS, 7LLF, 7LLZ, and 7LOS) (Table 1). Superposition of the ligand-bound structures shows all inhibitors utilized the same binding mode similar to GRL0617, including closure of BL loop and H bond between amide of inhibitor and D164 and Q269 (Osipiuk et al., 2021a; Gao et al., 2021; Ratia et al., 2008; Ahmad et al., 2021). The analysis of the representative co-crystal structures of XR8-24 and XR8-89 found that the azetidine ring extends into Site I to interact with side chain of E168 (Figures 5B,C) (Shen et al., 2021). The amide group of XR8-24 and XR8-89 is aligned closely with that of GRL0617 in SARS-CoV-2 PLpro (PDB: 7JRN) with the expected two H bonds between amide and the main chain of Q269 on the BL loop and with side chain of D164. In Site IV, the phenylthiophene group sits between P248 and side chain of Y268 at a similar position of naphthalene ring of GRL0617 (Figures 5B,C) (Shen et al., 2021). The thiophene extends further compared to naphthalene group of GRL0618 (Site V), where it takes part in van der Waals interactions with residues P248, Y264, and (Shen et al., 2021). The additional groups that derivatized from phenylthiophene have mostly poor electron density in crystal structures (Shen et al., 2021). Indeed, this region is open to solvent and authors conjectured that crystal packing forces might also contribute to it. However, the pyrrolidine ring of XR8-24 is better defined, with putative interaction with P248, G266, and Y265 (Figure 5B). In a plaque formation assay using the SARS-CoV-2 USA/WA1/2020 strain and Vero E6 cells. GRL0617 has an EC_50_ value of 21.7 ± 1.6 μM, whereas both XR8-23 and XR8-24 were significantly more potent than GRL0617 with EC_50_ at 2.8 ± 0.4 μM and 2.5 ± 1.9 μM, respectively. XR8-89 also demonstrated superior antiviral potency compared to GRL017, yet with higher EC_50_ value at 11.3 ± 1.6 μM. In this study, antiviral potency does not strictly correlate with the superior potency of this inhibitor in biochemical assays for unknown reasons. The lack of observable cytotoxicity for XR8-89 might indicate attenuated cell permeability as a cause of lower antiviral potency (Shen et al., 2021). No toxicity was observed under assay conditions in Vero E6 cells for these compounds at concentrations lower than 50 μM (Shen et al., 2021).

### GRL0667, Compound 3, Compound 15g, and Compound 15h

**FIGURE 6:**
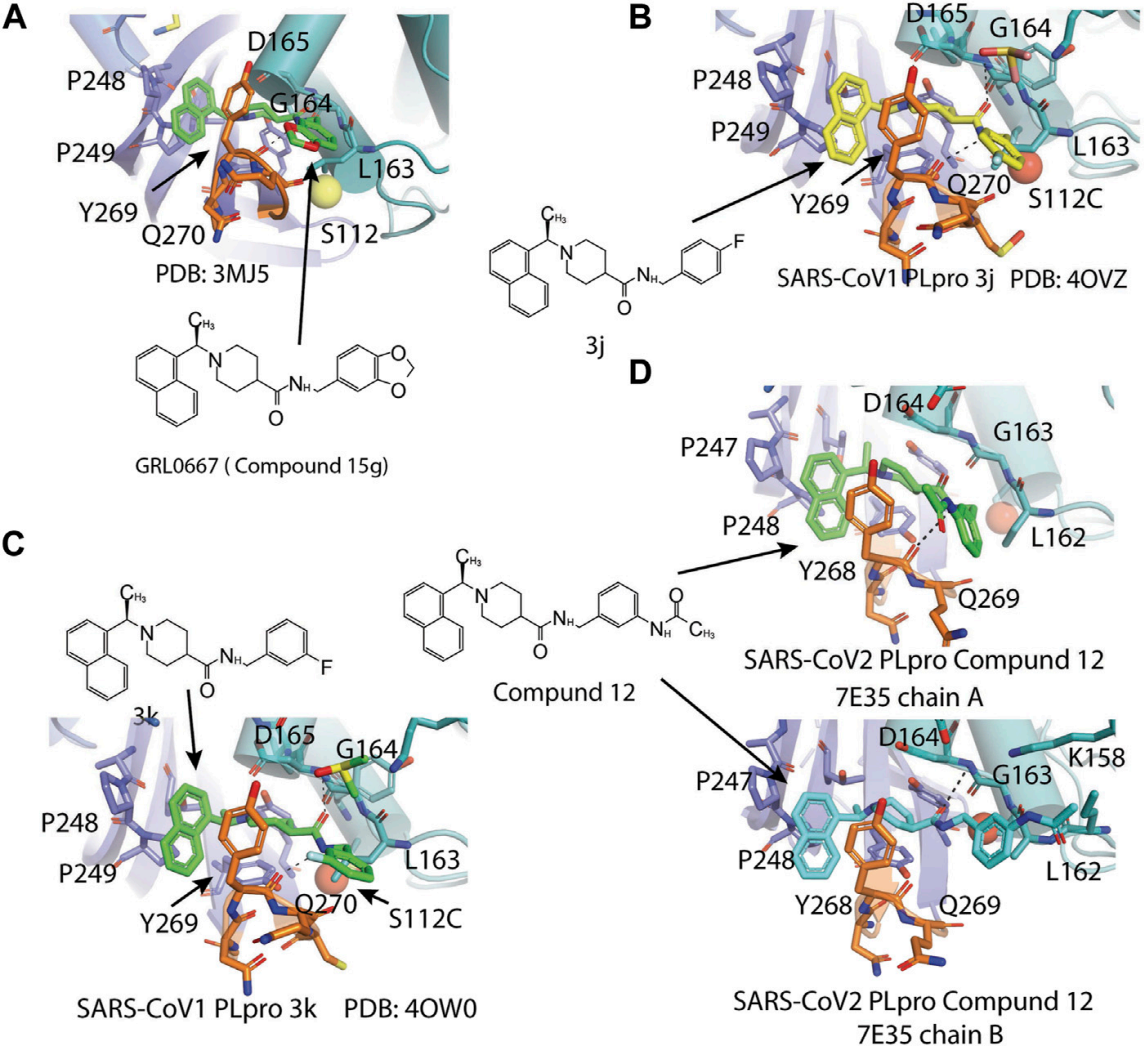
Close-up view of interaction between SARS-CoV-1 PLpro and inhibitors in crystal structures. PLpro is shown as cartoon with sticks representation shown for residues involving contact with inhibitors. Inhibitors are shown as sticks. Hydrogen bonds were labeled with dashed lines. **(A)** SARS-CoV-1 PLpro with GRL0667 (PDB: 3MJ5). **(B)** SARS-CoV-1 PLpro with 3j (PDB: 4OVZ). **(C)** SARS-CoV PLpro with 3k (PDB: 3OW0). **(D)** SARS-CoV-2 PLpro with compound 12 from the two copies in the asymmetric unit (PDB: 7E35).

Another lead compound 3 (6577871) was found *via* high-throughput screening of a diverse chemical library where GRL0617 was identified, but with lower potency IC_50_ = 59 μM (Table 2) (Ratia et al., 2008; Ghosh et al., 2010). Subsequent lead optimization efforts led to the design of potent inhibitor 15g (GRL0667, IC_50_ = 0.32 μM) which inhibited SARS-CoV-1 viral replication in Vero cells with an EC_50_ value of 9.1 μM, and its enantiomer 15 h has IC_50_ = 0.56 μM and similar antiviral potency (Table 2). The crystal structure shows that the naphthyl ring of 15g aligns in a similar fashion in the hydrophobic pocket formed by residues Y269, Y265, P248, P249, and T302, whereas the rest of the inhibitors exhibit different binding modes (Figure 6A) (note that GRL0617 was named compound 2 in this paper; PDB: 5MJ5) (Ghosh et al., 2010). The piperidyl group and the carboxamide group of 15g occupies similar position of methyl-aniline group of GRL0617, yet less bulky, so it allows side chain of Y269 to be slightly closer. The conformation of BL loop at Q270 is very different induced by binding of different inhibitors. Both the main chain and side chain of Q270 flip away from 15g, to make room for its benzodioxolane group that rests on the aliphatic region of Q270 side chain (Ghosh et al., 2010). The flexibility of BL loop is exploited by 15g.

**TABLE 2:** GRL0667-like inhibitors II.

Compound Name	Chemical Structure	IC_50_	EC_50_	References
6577871 (Compound 3)	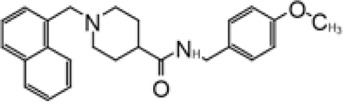	59.2 ± 7.8 μM (SARS-CoV-1)	—	Ghosh et al. (2010)
CP15g (GRL0667)	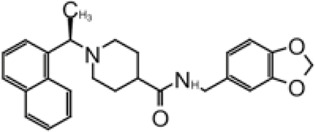	0.32 ± 0.01 μM (SARS-CoV-1)	—	
CP15 h	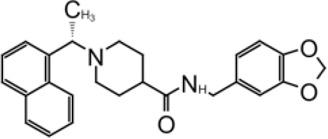	0.56 ± 0.03 μM (SARS-CoV-1)	—	
3J	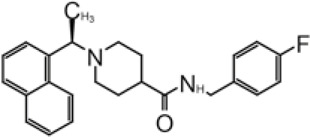	0.49 μM (SARS-CoV-1)	—	Báez-Santos et al. (2014b)
3K	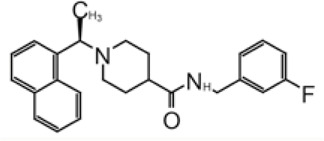	0.15 μM (SARS-CoV-1)	—	
rac5c	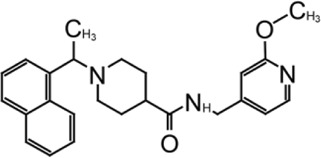	0.81 μM	—	Klemm et al. (2020)
Compound 12	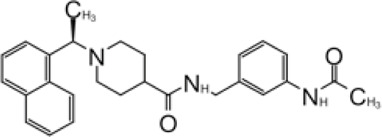	2.69 ± 0.34 μM	—	Shan et al. (2021)
Compound 14	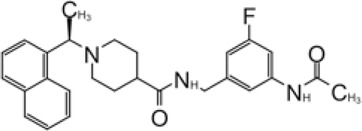	1.76 ± 0.06 μM	—	
Compound 18	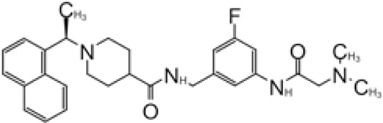	0.80 ± 0.29 μM	—	
Compound 19	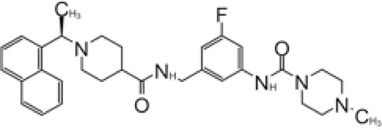	0.44 ± 0.05 μM	—	

In 2014, the same authors used SAR (structure–activity relationship) to show that adding additional groups to (R)-methyl group cause decreased potency, which is consistent with refinement of GRL0617 (Shen et al., 2021). Replacing benzodioxolane with 3-F-benzene or 4-F-benzene slightly increase potency (3j: 4-F IC_50_ = 0.49 μM; 3k: 3-F IC_50_ = 0.15 μM) (Table 2). The structural comparison of 3j and 3k with 15g shows that the binding modes to SARS-CoV-1 PLpro are almost identical with minor translation compared to GRL0617 (PDB: 4OVZ and 4OW0) (Figures 6B,C) (Báez-Santos et al., 2014b). Klemm et al. synthesized racemic forms of compounds reported in 2014: rac3j, rac3k, and rac5c (Table 2) (Báez-Santos et al., 2015; Klemm et al., 2020). Each compound had low or sub-micromolar inhibitory activity against SARS-CoV-2 PLpro. Rac5c is the best among the list, and it has IC_50_ value of 0.81 μM. It inhibited protease activity in the context of full NSP3 and inhibited viral replication at 11 μM concentration (Klemm et al., 2020).

Hengyue et al. prepared a series of reported SARS-CoV-1 PLpro inhibitors that share a naphthyl group with GRL0617 resembling GRL0667 (Shan et al., 2021; Ghosh et al., 2010; Báez-Santos et al., 2014b). These inhibitors showed better potency than GRL0617 in an *in vitro* fluorescence-based assay with IC_50_ values ranging between 2.6 and 4.3 μM. Authors solved the co-crystal structure of the SARS-CoV-2 PLpro C112S with compound 12 at 2.4 Å resolution (PDB: 7E35) (Table 2). In comparison with the apo structure, the BL loop adopts the same conformation as with complex with GRL0617 that both backbone of the BL loop and side chain of Y269 bends toward 12 (Figure 6D) (Gao et al., 2021; Ma et al., 2021a; Fu et al., 2021). Interestingly, the electron density maps indicated distinct binding modes of inhibitor for each asymmetric unit. In chain A, the phenyl ring of Y269 simultaneously engages with all three hydrophobic rings of 12, acting as a latch of the binding pocket. The carbonyl of Y269 forms an H bond with the backbone amide of 12 (Figure 6D). The derivatized cyclohexane extends into a small pocket formed by P248, R167, A247, and M209. This is different from compound 12 in chain B, where the same group is sandwiched by E162, L163, and the side chain of M209 from crystal packed molecule nearby (Shan et al., 2021). On the basis of the structure, further refinement of inhibitors was done with SAR. Piperidyl ring is tightly surrounded, so it is not an ideal candidate for refinement. Addition of 5-fluorine to benzene group of 12 potentially increased favorable contact with Q270, thereby increased potency of 14. Replacement of benzyl ring with piperidine ring were not successful, yet acetamide group extended by tertiary amine show increased potency: compounds 18 and 19 (Table 2). According to SPR results, 19 bound to SARS-CoV-2 PLpro with a *K*_d_ value of 2.6 mM, compared to that of GRL0617 at 10.8 mM. In addition, 19 is also shown not to inhibit DUBs at 10 μM and, at 10 mM 19, significantly inhibited SARS-CoV-2 PLpro in 293T cells and significantly recover the activation level of NF-κB that can be inhibited by PLpro. 19 could significantly inhibit SARS-CoV-2 replication even at 400 nM. At 10 mM, 19 did not show detectable cytotoxicity in hACE2-HeLa cells. 19 was the best in the series with an IC_50_ value of 182 nM and a therapeutic index (CC_50_/IC_50_) over 55 (Shan et al., 2021).

### Disulfiram

Disulfiram is a drug which was approved by the US FDA for use in alcohol aversion therapy (Lin et al., 2018). Disulfiram was first shown to inhibit SARS-CoV-1 PLpro at IC_50_ = 24.1 ± 1.8 μM by Lin et al., in 2018 (Table 3) (Lin et al., 2018). With a zinc-specific fluorophore, FluoZin-3, it was found that Zn^2+^ ion was released upon addition of disulfiram (Lin et al., 2018). The study by Karan et al. is in agreement with disulfiram functioning as a zinc ejector and confirmed the addition of disulfiram on PLpro with molecular weight calculated by mass spectrometry (Sargsyan et al., 2020). Another proposed mechanism of inhibition is the formation of a covalent adduct to catalytic cysteine, as BME treatment can partially restore PLpro activity inhibited by disulfiram (Lin et al., 2018). Efforts in obtaining the structure of disulfiram have not been successful. Lin et al. only observed BME like electron density projecting off SARS-CoV-1 PLpro C112 but not cysteines coordinating Zn^2+^, which supports the hypothesis that disulfiram inhibits PLpro by forming covalent adduct to catalytic cysteine. Smith et al. screened several libraries and found disulfiram and GRL0617 as the best leads (Smith et al., 2020), yet Gao et al. did not detect inhibition by disulfiram (Lin et al., 2018). The discrepancy may stem from different substrates used in assessing inhibitor efficacy or the presence of reducing reagents that neutralized the inhibitory effect. The inhibitory effect of disulfiram is greatly limited by the oxidation–reduction environment. Considering disulfiram is known to be promiscuous, the application and development of disulfiram for PLpro could be restricted.

**TABLE 3:** Other PLpro inhibitors.

Compound Name	Chemical Structure	IC_50_	EC_50_	References
Disulfiram	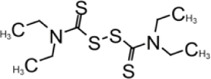	2 μM	—	Smith et al. (2020)
6-TG	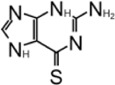	72 ± 12 μM	—	Fu et al. (2021)
Cryptotanshinone	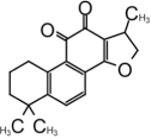	∼1–5 μM	∼1–5 μM	Yang et al. (2005); Park et al. (2012)
Dihydrotanshinone I	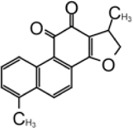	2.21 ± 0.10 μM	2.26 ± 0.11 μM	Yang et al. (2005)
Tanshinone IIA	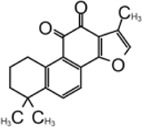	1.57 μM	—	Lim et al. (2021)
Tanshinone IIA sulfonate sodium	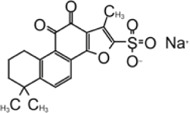	1.65 ± 0.13 μM	—	Xu et al. (2021)
Chloroxine	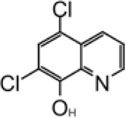	7.24 ± 0.68 μM	—	—
CPI-169	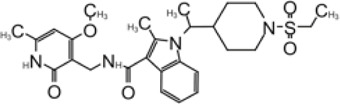	7.3 μM	—	Shen et al. (2021)
Ebselen	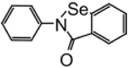	2.26 ± 1.05 μM	—	Weglarz-Tomczak et al. (2021)
VIR250	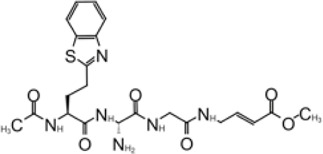	—	—	Rut et al. (2020a); Patchett et al. (2021)
VIR251	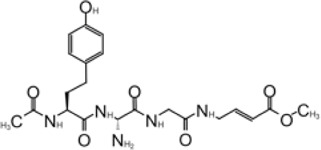	—	—	—
YM155	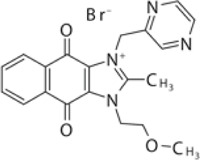	2.47 ± 0.46 μM	—	Zhao et al. (2021)
Jun9-13–7	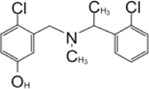	7.29 ± 1.03 μM	—	Ma et al. (2021a)
Jun9-13–9	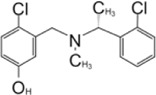	6.67 ± 0.05 μM	—	—

### 6-Thioguanine

6-Thioguanine (6-TG) is an FDA-approved drug that has been used in the clinic since the 1950s, originally for the treatment of childhood leukemias and subsequently for treatment of inflammatory bowel and Crohn’s disease (Bayoumy et al., 2020). Chou et al. discovered 6-TG as a reversible and slow-binding inhibitor for SARS-CoV-1 PLpro with IC_50_ = 5.0 ± 1.7 μM (Table 3) (Chou et al., 2008). Cheng found that 6-TG is an inhibitor for MERS PLpro (Cheng et al., 2015). Fu et al. used 6-TG, determined its potency against SARS-CoV-2 PLpro (IC_50_ = 72 ± 12 μM), and used it as a positive control for inhibitor screening (Fu et al., 2021). Gao et al. found that only 6-TG inhibited PLpro weakly with IC_50_ = 103.7 ± 49.4 μM (Gao et al., 2021). This preprint publication describes that an increasing concentration of 6-TG inhibited PLpro-mediated processing of the TAP-nsp123 WT polyprotein and blocked cleavage of ISG15 in HEK293T cells (Swaim et al., 2021). 6-TG inhibited viral replication in Vero-E6 cells with an EC_50_ value of 0.647 ± 0.374 μM, which is comparable to that of Remdesivir at 0.77 μM. 6-TG inhibited virus replication in Calu3 cells at a lower EC_50_, 0.061 ± 0.049 μM. 6-TG did not elicit significant cellular toxicity in either Vero-E6 or Calu3 cells (CC50 > 50 μM) (Swaim et al., 2021). Despite previous positive results in inhibition, in a recent study, 6-TG did not show binding in a TSA assay or inhibition in FlipGFP assay, therefore invalidating 6-TG as a PLpro inhibitor (Ma et al., 2021b).

### Tanshinone

Tanshinone is a class of compounds that was originally extracted from Salvia miltiorrhiza (Zhou et al., 2005). Tanshinone was identified as an inhibitor for SARS-CoV-1 PLpro in 2012 (Park et al., 2012). Authors extracted and tested a series of tanshinones with cryptotanshinone displayed the most potent inhibitory activity (IC_50_ = 0.8 μM) toward SARS-CoV-1 PLpro and weak inhibition for Mpro (IC_50_ = 226.7 ± 6.2 μM) (Table 3). The IC_50_ values demonstrated that the presence of naphthalene in tanshinone I (IC_50_ = 0.7 μM) provide a greater inhibitory effect than the other tanshinone derivatives. No detectable inhibition was observed for other proteases tested, including chymotrypsin, papain, and HIV protease (Park et al., 2012).

Zhao et al. (2021) found tanshinone while screening libraries against SARS-CoV-2 PLpro and determined that cryptotanshinone inhibited with an IC_50_ = 5.63 ± 1.45 μM and EC_50_ = 0.70 ± 0.09 μM, and tanshinone I has IC_50_ values of 2.21 ± 0.10 μM and EC_50_ = 2.26 ± 0.11 (Table 3) (Zhao et al., 2021).

Lim et al. found that dihydrotanshinone I inhibits SARS-CoV-2 PLpro with IC_50_ = 0.586 μM, in comparison to the IC_50_ values of 1.79 µM for GRL0617, 1.57 µM for tanshinone IIA, and 1.34 µM cryptotanshinone (Lim et al., 2021). Authors also found that dihydrotanshinone I has good specificity that it did not inhibit 3CLpro (Lim et al., 2021).

Yunxia et al. used ALKGG-AMC as substrate to screen a compound library with 1971 clinically approved compounds. Tanshinone IIA sulfonate sodium, a more water-soluble form of tanshinone was found to be a potent inhibitor. It has an IC_50_ value of 1.65 ± 0.13 μM, and the K_D_ value is 145 ± 8.5 μM (Table 3) (Xu et al., 2021). Tanshinone was found to directly interact with PLpro in biolayer interferometry (BLI) assay. Thermal shifting assay using SYPRO Orange found tanshinone IIA sulfonate sodium gently increased the thermo stability of PLpro by 1°C (Xu et al., 2021). As crystal structure of tanshinone with PLpro is not available, docking and molecular dynamics simulations were applied to indicate tanshinone IIA sulfonate sodium binds to P3–P4 sites and interacts with Y268, which is similar to the binding pocket of GRL0617 (Xu et al., 2021).

### Chloroxine

Along with tanshinone, chloroxine is also found to be a direct-interacting inhibitor for SARS-CoV-2 PLpro (Table 3) (Xu et al., 2021). It has IC_50_ value of 7.24 ± 0.68 μM, and the K_D_ value is 4.6 ± 0.29 μM. Thermal shifting assay using SYPRO Orange found mixing chloroxine compounds with PLpro increase Tm by 2.5°C. There is no crystal structure of chloroxine with PLpro available, so the mode of binding was illustrated by docking and molecular dynamics simulations (Xu et al., 2021). Chloroxine did not show stable binding to the active pocket but has a unique binding site at the PLpro-ISG15 binding interface, near residue R65. It was proposed that the binding of chloroxine could have a direct impact on interrupting the PLpro-ISG15 binding interface; however, it does not explain the inhibition of peptide-based substrate ALKGG-AMC as it is not expected to interact at S2 site (Xu et al., 2021). R65 is ∼38 Å away from the catalytic triad, so this binding mode awaits validation.

### CPI-169

A screening campaign using unbiased ChemDiv library (10,000-compound) and a biased, annotated TargetMol Bioactive library (5,370 compounds) for inhibitors only identified CPI-169 as a new inhibitor for SARS-CoV-2 PLpro (Table 3) (Shen et al., 2021). CPI-169 inhibits SARS-CoV-2 with an IC_50_ value of 7.3 μM. CPI-169 binds to PLpro moderately with K_D_ = 10.2 µM. In comparison, GRL0617 is slightly more potent with IC_50_ = 1.6 μM and K_D_ = 1.9 μM (Shen et al., 2021). Authors computationally docked to the BL loop of PLpro however were unable to obtain co-crystal structures. The SAR of CPI-169 is yet to be reported (Shen et al., 2021).

### YM155

**FIGURE 7:**
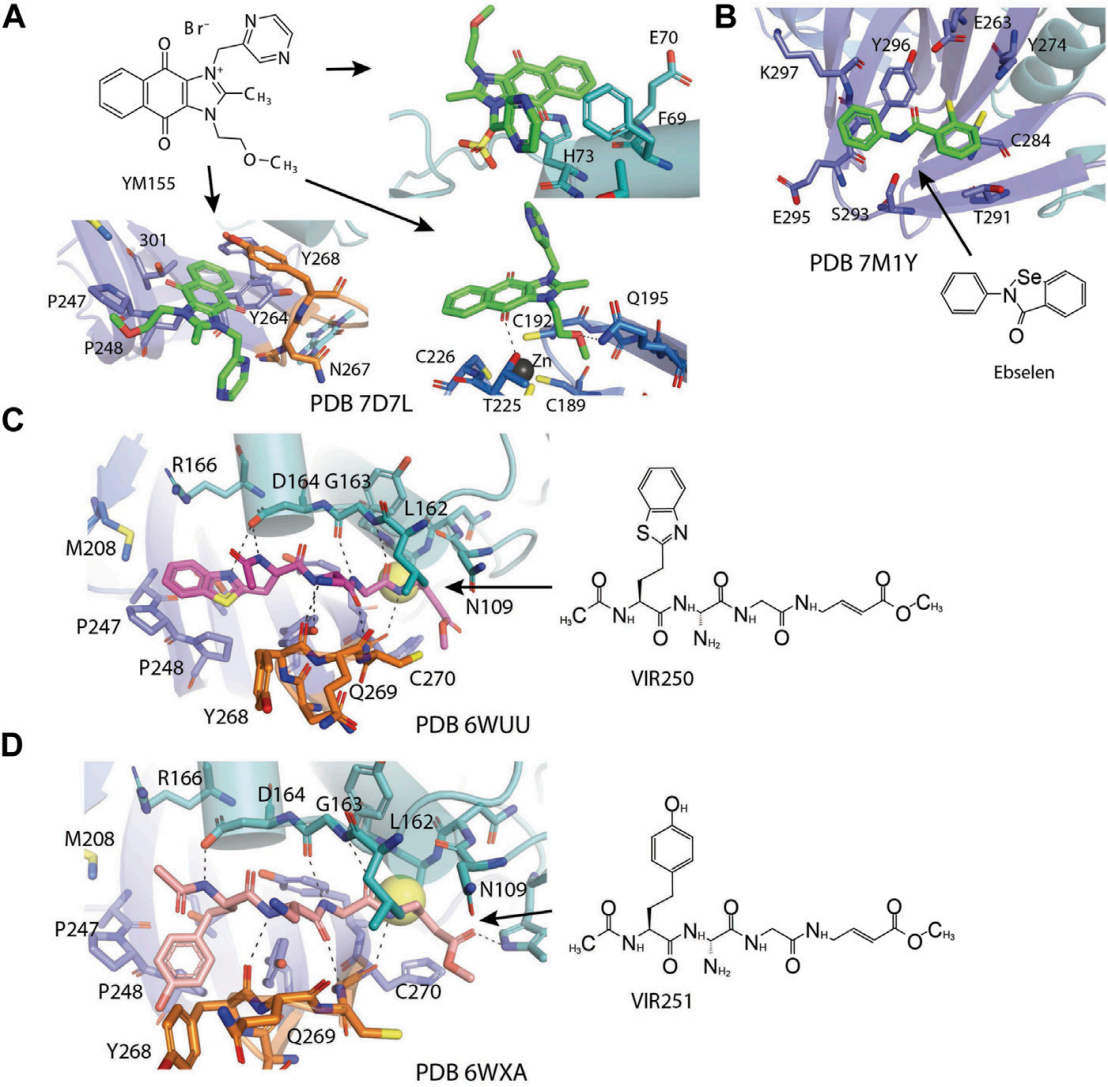
Close-up view of interaction between SARS-CoV-1 PLpro and inhibitors in crystal structures. PLpro is shown as cartoon with sticks representation shown for residues involving contact with inhibitors. Inhibitors are shown as sticks. Hydrogen bonds were labeled with dashed lines. **(A)** Three binding sites of YM155 on SARS-CoV-2 PLpro (PDB: 7D7L). **(B)** SARS-CoV-2 PLpro with ebselen (PDB: 7M1Y). **(C)** SARS-CoV-2 PLpro with VIR250 (PDB: 6WUU). **(D)** SARS-CoV-2 PLpro with VIR251 (PDB: 6WXA).

Zhao et al. (2021) found YM155 while screening 6,000 compounds from libraries consisting of approved drugs, drug candidates in clinical trials, and pharmacologically active compounds against SARS-CoV-2 PLpro (Table 3) (Zhao et al., 2021). YM155 is an antineoplastic drug in clinical trials, inhibited PLpro with an IC_50_ value of 2.47 μmol/L (Zhao et al., 2021). YM155 also exhibits strong antiviral activities in cell-based assays with an EC_50_ value of 170 nmol/L (Zhao et al., 2021). The crystal structure of YM155 with SARS-CoV-2 PLpro reveals three YM155 binding sites on SARS-CoV-2 PLpro (Figure 7A). The first YM155 molecule binds at the substrate binding pocket. Different from GRL0617, which occupies both P3 and P4 sites, YM155 only occupies P4 position (Figure 7A). The naphthoquinone aromatic group of YM155 forms hydrophobic interactions with the side chains of P248 and with the aromatic rings of Y264, Y268, and Y273. The plane of naphthoquinone group is tilted compared to naphthalene group of GRL0617. Importantly, binding of YM155 induced a unique conformation of BL loop. As previously described, BL loop closes upon GRL0617 binding, and Y268 flips toward GRL0617 and wedges between the two aromatic rings of GRL0617. When bound to YM155, Q269 flips away from body of the protease, making room for Y268 to shift toward the direction of thumb and Ubl domains. The side chain of Y269 forms *π*-stacking interaction with YM155, thus clamping the inhibitor to the protease (Zhao et al., 2021). On the basis of this binding site of YM155, the inhibitory effect can by rationalized by competition with the substrate.

The second YM155 binding site was observed near the thumb domain, interacting with F69 and H73 (Figure 7A). Interestingly, PLpro F69 is a critical residue at S2 involved in hydrophobic interactions to both distal Ub and NTD of ISG15 (Shin et al., 2020; Békés et al., 2016). YM155 here could function as a blocker for Ubl at S2 site. A third YM155 molecule is bound at the zinc-finger motif (Figure 7A). The methoxyethane group of the inhibitor inserts into the cleft, forming an H bond with Q195. A second H bond is formed between YM155 naphthoquinone group and T225. Binding of YM155 distorted the region containing C224 and C226, compared to apo structure (PDB: 7D7L) (Zhao et al., 2021). The Finger domain is important for the proteolytic and deubiquitinating activity of PLpro, so this YM155 binding site may also significantly contribute to its inhibitory effects (Herold et al., 1999; Klemm et al., 2020). The inhibitory effects of three individual binding sites for YM155, and SAR of YM155 derivatives need to be investigated.

### Ebselen

Ebselen is a low–molecular weight organoselenium drug that has low toxicity to use in humans (Table 3) (Azad and Tomar 2014). Karen et al. found that ebselen inhibits PLpro with the similar mechanism of disulfiram that it covalently adds to cysteines of SARS-CoV-2 PLpro and ejects Zn^2+^. It has an IC_50_ value of 0.67 ± 0.09 μM measured by fluorogenic peptide substrate (Sargsyan et al., 2020). Ebselen is a dual inhibitor for both PLpro and Mpro with higher potency toward Mpro (Jin et al., 2020a; Zmudzinski et al., 2020; Weglarz-Tomczak et al., 2021). Jin et al. proposed a reaction mechanism for ebselen inhibiting Mpro, and the same mechanism might account for its inhibition to PLpro (described in the Mpro section) (Jin et al., 2020a). In our preprint publication on BioRxiv, the best inhibitor among a series of ebselen derivatives, Compound 7, has an IC_50_ value of 0.58 ± 0.04 μM. However, Gao et al. found ebselen not to be inhibitory in their assay (Table 3) (Gao et al., 2021). The discrepancy may stem from different substrates used for assessing inhibitor efficacy or the assay condition, e.g., reducing reagent.

The mechanism of inhibition by ebselen to Mpro is by the formation an adduct at the catalytic cysteine to form a seleno sulfide bond. (Amporndanai et al., 2021). Both Mpro and PLpro are cysteine proteases, and ebselen may inhibit PLpro in the same way. However, the analysis of a recent unpublished PDB accession 7M1Y found that, when crystallized with PLpro C111S mutant, weak electron density demonstrates ebselen bound at a shallow pocket on palm domain. Selenium phenyl group is surrounded by side chains of residues E263, K274, C284, T291, and Y296, and at the other end, phenyl group is lined by backbone of E295 and Y296 (Figure 7B). Ebselen inhibited viral replication with EC_50_ = 4.67 μM, which could a combined effect of targeting both Mpro and PLpro (Jin et al., 2020a).

### Peptide Inhibitors: VIR250 and VIR251

Hybrid combinatorial substrate library (HyCoSuL) is a combinatorial library of tetra-peptides containing natural and unnatural amino acid mixtures at the P4–P2 positions, a fixed amino acid at the P1 position, and an ACC (7-amino-4-carbamoylmethylcoumarin) fluorescent tag occupying the P1’ position (Drag et al., 2008; Rut et al., 2020b). Once the peptide is recognized and cleaved by a protease, the ACC is released and produces a readable fluorescence signal. This method was used to investigate DUBs’ activity (Drag et al., 2008; Rut et al., 2020b). A series of tetrapeptide-ACC including natural and unnatural amino acid residues was designed and synthesized, and the best amino acid composition to target SARS-CoV-2 PLpro was determined (Poreba et al., 2017). The preferred substrates [Ac-hTyr-Dap-Gly-Gly-ACC, VIR251, and Ac-Abu (Bth)-Dap-Gly-Gly-ACC, VIR250] were converted into inhibitors by exchanging the fluorescent tag to a vinylmethyl ester (VME) group (Table 3) (Rut et al., 2020a). Both VIR250 and VIR251 exhibit high selectivity and robust inhibition toward both SARS-CoV-1 PLpro and SARS-CoV-2 PLpro, whereas no inhibition of human DUB (UCH-L3) was observed in biochemical assay and cell lysate-based assay (Rut et al., 2020a). This high specificity is important for drug discovery purposes.

The crystal structures of VIR250 and VIR251 in complex with both SARS-CoV-1 and SARS-CoV-2 PLpro in combination were determined, in collaboration with our lab, for the purpose of understanding the binding mode and future refinement of the inhibitors (Figures 7C,D) (Rut et al., 2020a; Patchett et al., 2021). As expected, the catalytic C111 is covalently linked to the *ß* carbon of the vinyl group of the VME warheads of inhibitors with thioether linkages. Both inhibitors occupy the P1–P4 pockets of SARS-CoV-2 PLpro. P1 and P2 sites of inhibitors are Gly residues, which are the same as Ub and ISG15. At P3, inhibitors have Dap, which is an unnatural amino acid residue, whereas in Ub, it is Arg, and, in ISG15, it is Pro. Dap of VIR250 participates in a backbone–backbone H bond with G271 and Y268, whereas that of VIR251 engages in the backbone–backbone H bond with Y268 (Figures 7C,D). More importantly, whereas P1–P3 residues engage very similar contacts with PLpros of both species, P4 residues show significant diverse conformations. When crystallized with SARS-CoV-2, VIR250 P4 Abu (Bth) projects toward Finger domain and engages in a network of van der Waals interactions with M208, P247, P248, and T301, this contrasts its conformation when crystallized with SARS-CoV-1, which the side chain flips about 90° pointing in the direction of palm domain. Interestingly, SARS-CoV-2 VIR251 hTyr at the P4 position projects toward the palm domain, which is same as SARS-CoV-1 VIR250 P4 side chain, and opposite of SARS-CoV-2 VIR251 P4, whereas SARS-CoV-2 VIR250 P4 side chain is pointing to similar direction as SARS-CoV-2 VIR251 P4 (Figures 7C,D).

The significant freedom of P4 site is consistent with the observation that P1–P2 sites are narrow and less accessible, P3 site is half exposed, whereas P4 site is broad and well solvent exposed. The P3–P4 sites are exploited by both small-molecule inhibitors and peptide inhibitors. The different conformations of P4 side chain of inhibitors are accompanied by slight shift of the BL loop and different rotamers of key residues including Y268 and Q269. Interestingly, when compared with the GRL0617-bound PLpro structure, the side chains of P4 of inhibitors partially overlap with naphthalene group from GRL0617 (Figures 7C,D). With the previous success in replacing naphthalene with longer biaryls (Shen et al., 2021), it is possible to elongate the side chain of P4 position of peptide inhibitor in future refinement.

### Jun9-13-7 and Jun9-13-9

Ma et al. found two new inhibitors Jun9-13-7 and Jun9-13-9 screening against the Enamine 50K diversity compound library and subsequent lead optimization (Table 3) (Ma et al., 2021a). Jun9-13-7 and Jun9-13-9 had IC_50_ values of 7.29 ± 1.03 and 6.67 ± 0.05 μM, respectively (Ma et al., 2021a). The two inhibitors also increased the thermal stability of SARS-CoV-2 PLpro by 2.98 ± 0.09°C and 2.18 ± 0.29°C. The inhibition by these two hits is slightly weaker than GRL0617 tested under same conditions: IC_50_ value of 2.05 ± 0.12 μM.

Subsequent lead optimization led to the discovery of several inhibitors with sub-micromolar potency in the enzymatic assay. Among them, Jun9-75-4 was the most potent PLpro inhibitor with an IC_50_ value of 0.62 μM, a 10-fold increase compared to original hit, and three-fold more potent than GRL0617. Without a structure of the new inhibitors with PLpro, the authors used molecular dynamics method to analyze how the inhibitors interact with SARS-CoV-2 PLpro (Ma et al., 2021a).

## Structural Biology of SARS-CoV-2 Main Protease

**FIGURE 8:**
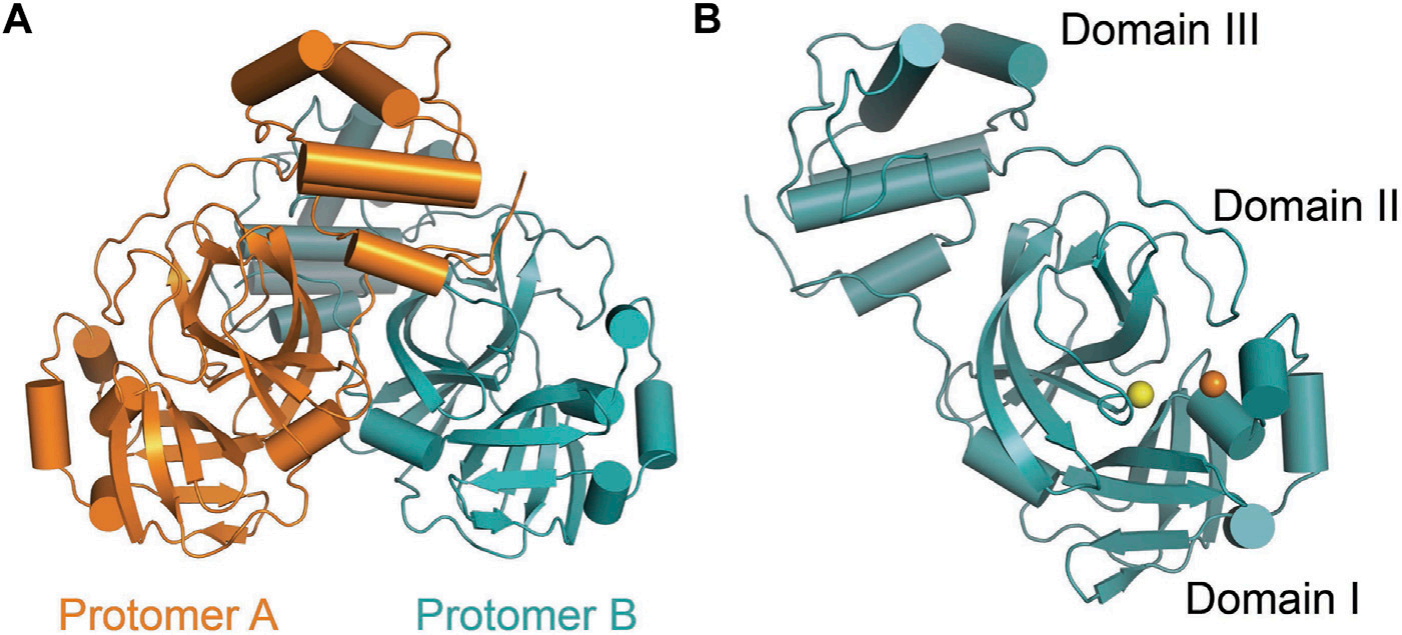
Three-dimensional structure of SARS-CoV-2 Mpro. **(A)** One protomer of the dimer is shown in orange, and the other one is shown in teal (PDB: 6LU7). **(B)** Cartoon representation of the crystal structure of one protomer. The amino acid residues of the catalytic site are indicated as yellow sphere for Cys145 and orange sphere for His41.

SARS-CoV-2 Mpro (nsp5, also referred to as 3CLpro) is a cysteine protease that is widely conserved among coronaviruses. Mpro operates at the recognition sequence Leu-Gln↓ (Ser, Ala, Gly) (↓ marks the cleavage site) to mediate the maturation cleavage of polyproteins nsp4–16 during virus replication. There is no known human protease with a specificity for Gln at the cleavage site of the substrate (Zhang et al., 2020a). This feature along with its essential function in viral cell cycle makes Mpro a promising target for COVID-19 treatment development. The active version of Mpro is a homodimer, and each protomer is comprised of three domains (domains I, II, and III) (Figure 8). The domains I (residues 8–101) and II (residues 102–184) consist of antiparallel *ß*-barrels, and together, they form the chymotrypsin-like structure. The domain III (201–306), which is mostly composed by *α*-helices, is responsible for the dimerization process. SARS-CoV-2 Mpro has 96% primary sequence identity to that from SARS-CoV-1. A notable difference in SARS-CoV-2 Mpro is the mutation of T285 and I286 to Ala and Leu, respectively, when compared to that from SARS-CoV-1 (Zhang et al., 2020b). These changes keep the two domains III closer, leading to an increase in catalytic turnover.

**FIGURE 9:**
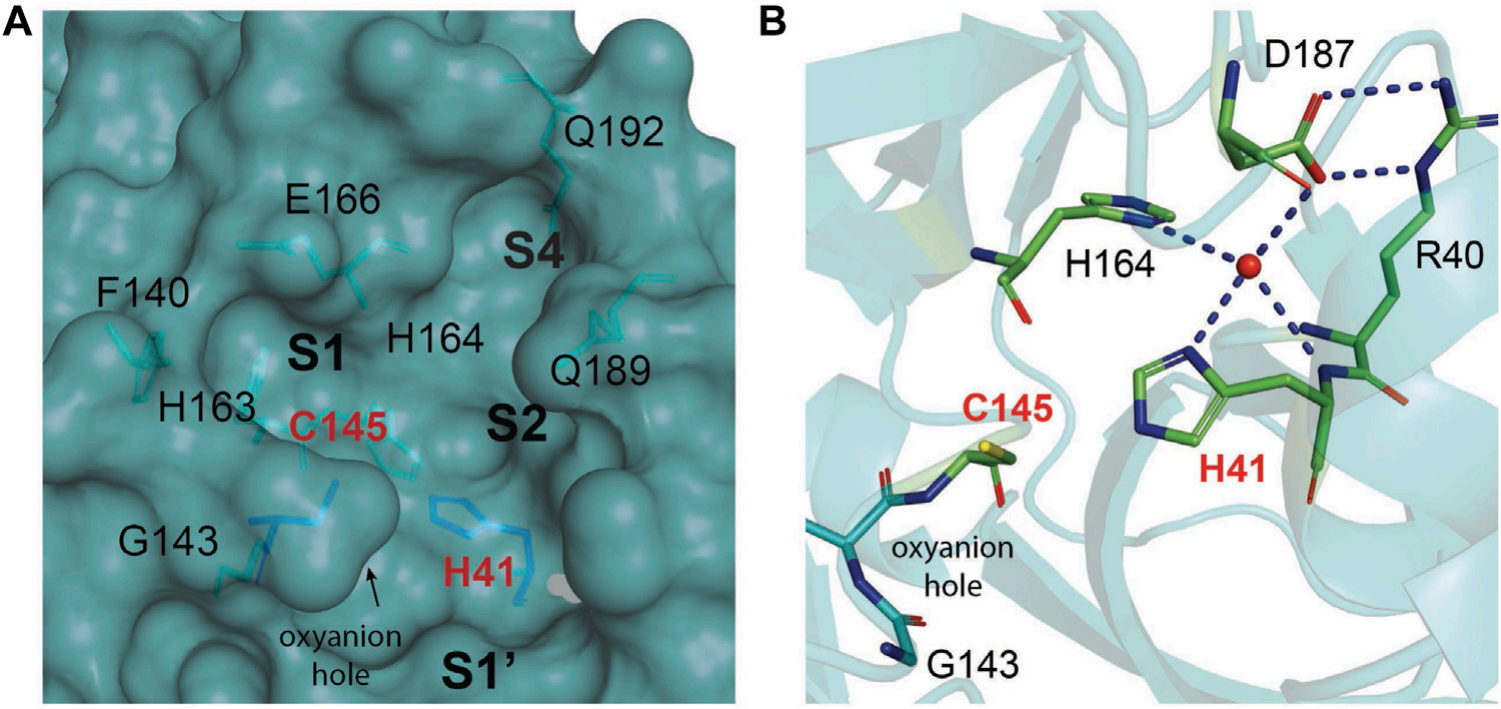
The substrate-binding cleft located between domains I and II of Mpro. **(A)** The active site cavity is located on the surface of Mpro. Subsites S1, S2, and S4 are shaped into well-formed binding pockets. The catalytic dyad is highlighted in red with the residues that flank the cavity. The oxyanion hole created by residues 140–144 is highlighted. **(B)** A close-up view of the catalytic site cavity in which the catalytic residues (Cys145 and His41) are highlighted in red. The catalytic water molecule is shown as a red sphere. Hydrogen bonds are shown as black dashed lines.

The substrate-binding pocket lies in the cleft between domains I and II. The active site of the enzyme consists of four pockets (S1’, S1, S2, and S3), with the S1 pocket containing a catalytic dyad (Figure 9A). This catalytic dyad is composed of the C145 and H41 residues. The absence of the standard third catalytic element is compensated by the presence of a buried water molecule, which forms H bonds with the residue of H41 and the surrounding amino acids (Figure 9B) (Anand et al., 2003; Kneller et al., 2020a; Kneller et al., 2020b; Citarella et al., 2021). The active site of Mpro is favored by strong H bond interactions with an “oxyanion hole” formed by G143, S144, and C145 (Świderek and Moliner, 2020). The stabilization of the oxyanion by the H bonds in the transition state should contribute to the catalytic activity (Simón and Goodman, 2010). Another water molecule is located within the active site of the enzyme and establishes H bonds with F140, H163, and E166, further stabilizing the oxyanion hole.

**FIGURE 10:**
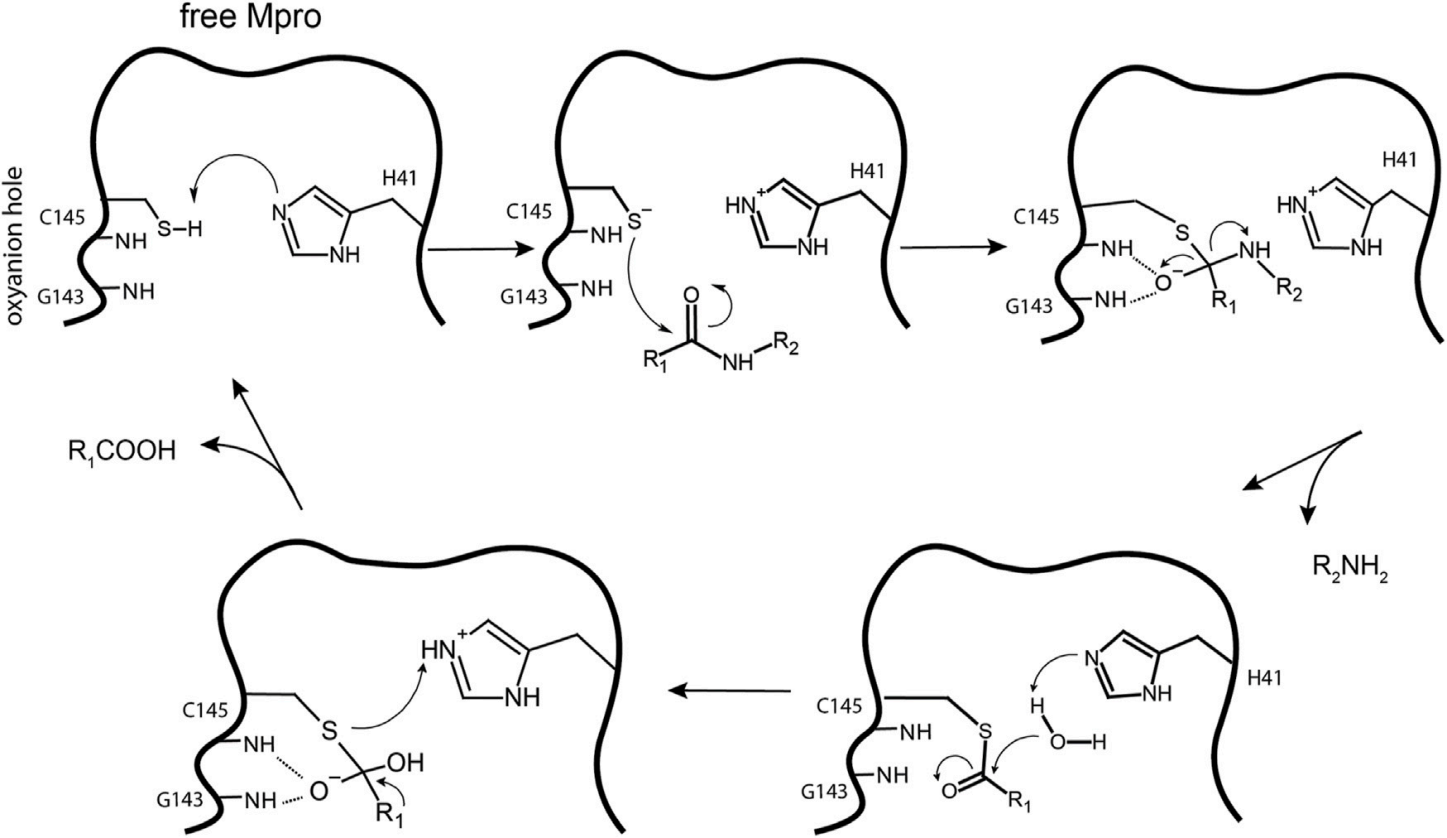
Hydrolysis mechanism of SARS-CoV-2 Mpro. In the free state, H41 of Mpro deprotonates the thiol of C145. The next step is nucleophilic attack by the deprotonated C145 sulfur on the peptide carbonyl carbon. Then, a fragment of the substrate (R2) is released, and the histidine is restored to its deprotonated form. The new carboxyl-moiety undergoes nucleophilic attack by water, which results in H41 becoming protonated. The thioester bond is subsequently hydrolyzed to generate a C-terminus on the remaining substrate fragment while regenerating the free enzyme.

The thiol group of C145 is responsible for hydrolysis. The initial step in the process is deprotonation of Cys-thiol and followed by nucleophilic attack of resulting anionic sulfur on the substrate carbonyl carbon. In this step, a peptide product is released, whereas H41 is restored to its deprotonated form. The resulting thioester is hydrolyzed to release a carboxylic acid, and the free enzyme is regenerated in the final step (Figure 10) (Pillaiyar et al., 2016). The interaction of the amino-terminus (N-terminus) of one protomer with domain II of the other *via* H bonding helps shape the S1 pocket of the active site (Zhang et al., 2020a; Zhang et al., 2020b). Simulations showed that the active site residues and the substrate binding pocket are not in the proper conformation for catalysis in the monomers (Chen et al., 2006). Therefore, the dimer is the active form, whereas the monomer is inactive (Goyal and Goyal, 2020). Compounds that can interfere with the dimeric interface may act as potent inhibitors.

## Inhibitors Against Main Protease

Although there is currently a good clinical candidate for COVID-19, focus should not be taken away from the study of other potential Mpro inhibitors. The inhibitor studies this past year and half could further help design new treatments for COVID-19 in addition to preparing for future coronavirus outbreaks. These studies have included drugs developed as treatment for other viruses and new compounds specific for coronaviruses. Because of their success in the treatment, the repurposing of FDA-approved drugs allows speeding up the experimental phases of a new therapy, since safety studies have already been validated. Several inhibitors have been developed against SARS-CoV-2 Mpro, and these are typically peptidomimetics that mimic natural peptide substrates (Jacobs et al.,2013); Tian et al., 2021; Jacobs et al., 2013; Tian et al., 2021). The warheads mainly contain Michael receptors, aldehydes, and different types of ketones, which covalently bind to the C145 residue in Mpro to exert an inhibitory effect. The *α*-ketoamide warhead is sterically more versatile than other warheads because it features two acceptors for H bonds from the protein, whereas the other warheads have only one such acceptor. Here, we focus on key interactions of some of the most promising results, which have become the basis for further derivatization (Table 4).

**TABLE 4:** Mpro inhibitors.

Compound Name	Chemical Structure	IC_50_	EC_50_	References
PF-07321332	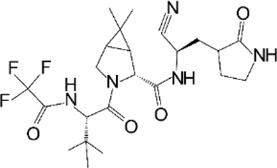		74.5 nM	Owen et al. (2021)
Ebselen	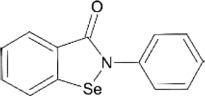	0.67–2.1 μΜ	4.67 μM	Jin et al. (2020a); Ma et al. (2020a); Banerjee et al. (2021)
MR6-31–2	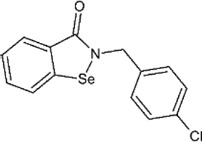		1.8 µM	Amporndanai et al. (2021)
Boceprevir	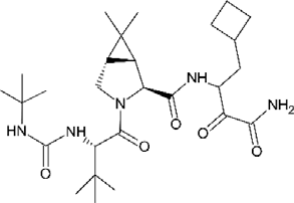	1.59–8 μM	1.90–15.57 μM	Ma et al. (2020b); Fu et al. (2020); Oerlemans et al. (2021)
GC-376	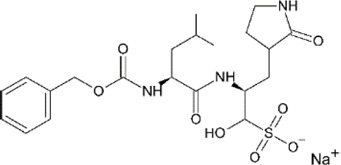	0.03–0.19 µM	0.7–0.92 µM	(Ma et al. (2020b); Fu et al. (2020); Vuong et al. (2020)
GC-376 derivative 2c	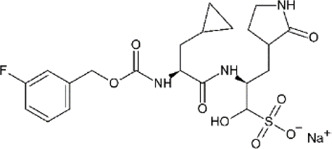	0.07 µM	0.57 µM	Vuong et al. (2021)
GC-376 derivative 2d	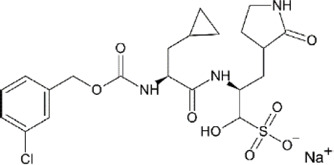	0.08 µM	0.7 µM	Vuong et al. (2021)
N3 derivative 11a	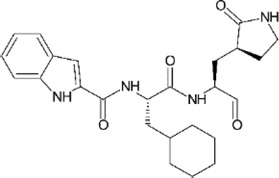	0.053 µM	0.53 µM	Dai et al. (2020)
N3 derivative 11b	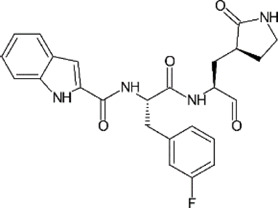	0.040 µM	0.72 µM	Dai et al. (2020)
Calpeptin	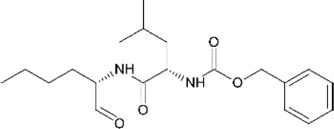	10.69 µM	72 nM	Ma et al. (2020b); Günther et al. (2021)
Carmofur	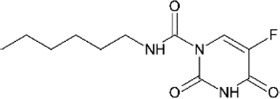	1.82 µM	24.30 μM	Jin et al. (2020b)
Pelitinib	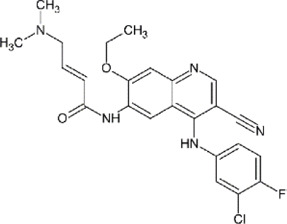		1.25 μM	Günther et al. (2021)
ML188 derivative 23R	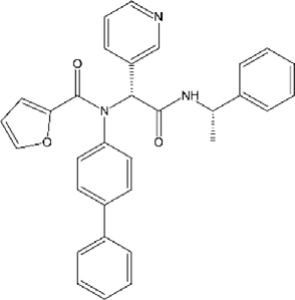	0.2 ± 0.01 μM	3.03 μM	Kitamura et al. (2021)
Perampanel derivative 26	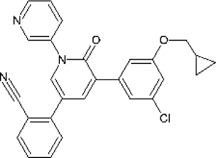	0.170 μM	0.98 μM	Zhang et al. (2021)

### PF-07321332

To date, PF-07321332 is one of two orally available COVID-19 antiviral clinical candidates (along with Molnupiravir). The structure of this inhibitor was revealed by Pfizer at the American Chemical Society Spring 2021 meeting (Halford, 2021). Ritonavir is commonly used in conjunction with other protease inhibitors to inhibit cytochrome P450-3A4. Co-administration with a low dose of ritonavir is expected to help slow the metabolism, or breakdown, of PF-07321332, allowing it to remain active in the body for longer periods of time at the higher concentrations needed to help combat the virus (Zeldin and Petruschke, 2004). In September 2021, they announced the start of the phase 2/3 trial to evaluate the prevention of illness in adults living in the same household as someone with COVID-19 (Pitts, 2021). Recently, Pfizer disclosed that PAXLOVID™ (a PF-07321,332/ritonavir combination) can reduce risk of hospitalization or death by 89% in non-hospitalized adult patients with COVID-19, who are at high risk of progressing to severe illness when administered within 3 days of symptom onset (Pfizer, 2021).

**FIGURE 11:**
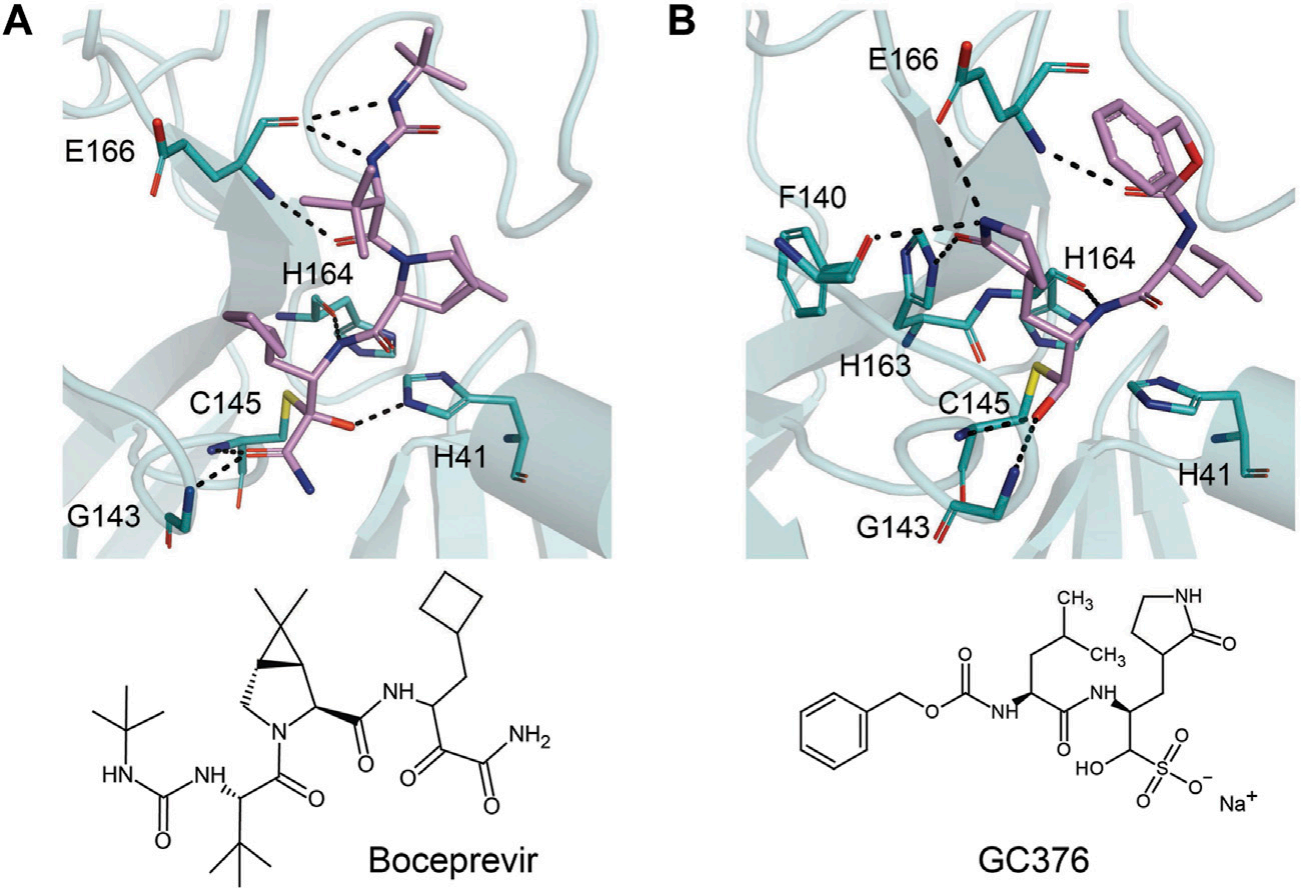
Close-up view of the interactions between SARS-CoV-2 Mpro with Boceprevir and GC376. Close-up view of interaction between SARS-CoV-2 Mpro and inhibitors in crystal structures. Mpro is shown as cartoon representation, with residues involved in the interaction shown as sticks. Inhibitors are shown as violet sticks. Hydrogen bonds were labeled with dashed lines. **(A)** Boceprevir (PDB: 7C6S). **(B)** GC376 (PDB: 7C6U).

The prodrug PF-07321332 was specifically developed to be administered orally to block SARS-CoV-2 Mpro activity. It was derived from PF-00835231, a phase I clinical candidate (prodrug PF-07304814) originally developed by Pfizer in 2002–2003 against SARS-CoV-1 (Hoffman et al., 2020). Owen et al. reported improved antiviral activity (EC_50_ = 74.5 nM) compared to the parent compound (EC_50_ = 231 nM). PF-07321332 shares the dimethylcyclopropylproline and tert-leucine features of Boceprevir, an inhibitor developed for the Hepatitis C Virus (HCV) NS3 protease (described further below). Molecular simulations proposed that the new inhibitor PF-07321332 interacts similarly as Boceprevir (Figure 11A) with the additional feature of an H bond between the pyrrolidone group and H163, similar to the PF-00835231 interaction (Hoffman et al., 2020; Pavan et al., 2021). The co-crystal structure of PF-07321332 is set to be released soon (PDB: 7RFW) (Owen et al., 2021). The preprint reveals that the inhibitor forms H bonding interactions with Q189, E166, and H163. The P1’ nitrile forms a covalent thioimidate adduct with the catalytic C145, which was confirmed to be reversible by recovery of Mpro activity after dilution of the complex (Owen et al., 2021).

### Ebselen

Ebselen is an organoselenium molecule that can function as a glutathione peroxidase and peroxiredoxin mimic (Nakamura et al., 2002). It has been shown to form a seleno-sulfide bond with thiol groups of cysteine on several proteins, which results in anti-inflammatory, anti-microbial, and neuroprotective effects (Amporndanai et al., 2021). Ebselen was identified in a high-throughput screen as a potential hit of SARS-CoV-2 Mpro inhibitor with an IC_50_ between 0.67 and 2.1 μΜ (Jin et al., 2020a; Ma et al., 2020a). Amporndanai et al. assessed derivatives for their inhibition of SARS-CoV-2 Mpro and anti-coronaviral activity (Amporndanai et al., 2021). Two of these ebselen-based selenium compounds exhibit greater inhibitory effectiveness against Mpro enzyme and SARS-CoV-2 replication. It is proposed that the ebselen-enzyme drug protein adduct is hydrolyzed by the conserved water in the catalytic pocket. Co-crystallographic structure of Mpro grown with ebselen and its derivative MR6-31–2 showed an electron density coordinating to C145, which is likely to be selenium due to its size and its absence in compound-free Mpro crystals. MR6-31–2 is nearly three times more effective with an EC_50_ value of 1.8 μM (ebselen EC_50_ = 4.67 μM) (Banerjee et al., 2021). As mentioned in the previous sections, ebselen and its derivatives have been shown to bind and inhibit PLpro, and this dual action inhibition may be the source of the potent antiviral activity.

### Boceprevir

Boceprevir is an FDA-approved serine protease inhibitor to treat HCV infection. Similar to the coronavirus Mpro proteases, cleavage of the HCV polyprotein by the viral NS3 protease releases functional viral proteins essential for viral replication (Tomei et al., 1993). It was reported that the ketoamide group of Boceprevir can bind covalently to the catalytic S139 of HCV NS3 protease (Malcolm et al., 2006).

This drug was screened alongside other viral protease inhibitors and has been shown to inhibit the enzymatic activity of Mpro with an IC_50_ value of 4.13 μM and has an EC_50_ value of 1.90 μM against the SARS-CoV-2 virus (Ma et al., 2020b). In the Mpro–Boceprevir complex structure (PDB: 6ZRU and 7C6S) (Oerlemans et al., 2021; Fu et al., 2020), the nucleophilic C145 in Mpro forms a C–S covalent bond with the keto carbon of Boceprevir, and the resulting hydroxyl group forms an H bond with the side chain of H41 and stabilizes this conformation (Figure 11A). Boceprevir also interacts with the oxyanion hole, with the oxygen of the *α*-ketoamide forming H bonds with the main chain amides of C145 and G143. The tert-butyl urea group orients into the S4 pocket and is stabilized by several H bonds with the main chain oxygen of E166 and hydrophobic interactions with the side chains of M165, Q192, L167, and P168. The cyclobutylalanine P1 residue has no interaction with the S1 subsite (Fu et al., 2020).

### GC376

GC376 is a bisulfite adduct prodrug of the corresponding aldehyde, GC373, which strongly inhibits the Mpro of several coronaviruses, including SARS-CoV-2 (IC_50_ value of 0.03–0.19 µM and EC_50_ value of 0.92 µM) (Ma et al., 2020b; Vuong et al., 2020). These drugs are able to block virus replication in cell culture and are well tolerated by various cell lines in cellular cytotoxicity tests (Ma et al., 2020b), indicating that they are good candidates as antivirals for the treatment of COVID-19. An NMR study supports the proposal that, in aqueous solutions, diastereomers of GC373 and GC376 exist in a dynamic stereochemical equilibrium, with only the correct aldehyde isomer binding as a single hemithioacetal in the active site of Mpro (Figure 11B) (Vuong et al., 2021). The crystal structure of SARS-CoV-2 Mpro with GC376 indicates the bisulfite group is readily removed and the aldehyde form (GC373) covalently bonds to catalytic C145 (PDB: 7C6U). The ring at the P1 position of GC376/GC373 fits into the S1 pocket and has H bonding interactions with the carboxyl group of E166, the carbonyl group of F140, and the imidazole of H163. Inhibitor binding is further stabilized by the leucine of GC376 interacting with the hydrophobic S2 subsite and the carbonyl in P3 forming an H bond with the backbone amide of E166. In the SARS-CoV-2 dimer, the thioacetal hydroxide H bonds to “oxyanion hole” formed by the backbone amides of G143, S144, and C145, resulting in the (S)-configuration seen with other aldehydes (11a and 11b) (Vuong et al., 2020), but in the instance of three protomers per asymmetric unit, the third copy of GC376 was able to able bind in the (R)-configuration with the hydroxide H bonding to H41 (Ma et al., 2020b).

GC376 has been shown to be more potent than Boceprevir; however, possible side effects in animal use could limit its use for less than 2 weeks (Fu et al., 2021). Improvements by modification of the chemical structure of GC376 resulted in a number of compounds with improved binding characteristics and nanomolar inhibition of SARS-CoV-2 Mpro. The optimal modification for the P2 site of the inhibitor was a cyclopropyl moiety. Inhibitors 2c and 2d emerged as key compounds for Mpro enzyme inhibition with better IC_50_ and cellular EC_50_ values compared to the parent inhibitor GC376 (2c: IC_50_ = 0.07 µM, EC_50_ = 0.57 µM; 2d: IC_50_ = 0.08 µM, EC_50_ = 0.7 µM) (Vuong et al., 2021).

### N3

**FIGURE 12:**
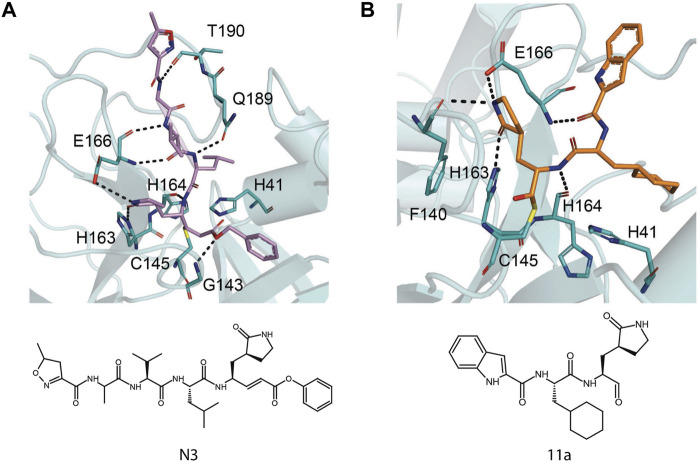
Close-up view of the interactions between SARS-CoV-2 Mpro with N3 and its derivative 11a. Close-up view of interaction between SARS-CoV-2 Mpro and inhibitors in crystal structures. Mpro is shown as cartoon representation, with residues involved in the interaction shown as sticks. Inhibitors are shown as sticks. Hydrogen bonds were labeled with dashed lines. **(A)** N3 (PDB: 6LU7), violet. **(B)** 11a (PDB: 6LZE), orange.

A mechanism-based inhibitor, N3, which was identified by a structure assisted optimization program, can specifically inhibit Mpro from multiple coronaviruses, including SARS-CoV-1and MERS-CoV (Yang et al., 2005; Jin et al., 2020a). N3 is an irreversible inhibitor that forms adduct with the catalytic cysteine by Michael addition of the C*β* atom of the vinyl group. The crystal structure with SARS-CoV-2 Mpro shows that N3 binds to the active site in an extended conformation (PDB: 6LU7) (Jin et al., 2020a). The peptidyl backbone of the inhibitor forms an antiparallel sheet with residues 164–168 and residues 189–191 on the other. The P1’ benzyl ester forms van der Waals interactions with T24 and T25. The γ-lactam ring at P1 inserts into S1 subsite and H bonds with H163. The side chain of leucine at P2 inserts deeply into the hydrophobic S2 subsite formed by H41, M49, Y54, M165, and D187. The side chain of valine at P3 is solvent exposed. The side chain of alanine at P4 occupies the hydrophobic pocket formed by the side chains of M165, L167, F185, and Q192 and the main chain of Q189. P5 makes van der Waals contacts with P168 and residues 190–191 (Figure 12A). N3 displayed inhibition against SARS-CoV-2 with an EC_50_ value 16.77 μM (Jin et al., 2020a).

### 11a and 11b

The co-crystal structure of N3 with SARS-CoV-2 has been the model for many structure-guided designs. Among those is the study by Dai et al. (2020). The aldehyde compounds 11a and 11b showed good inhibitory activity against SARS-CoV-2 Mpro (11a: IC_50_ = 0.053 ± 0.005 µM, 11b: IC_50_ = 0.040 ± 0.002 µM) and good anti–SARS-CoV-2 infection activity in cell culture, with EC_50_ values of 0.53 ± 0.01 µM and 0.72 ± 0.09 µM, respectively, by plaque assay. The crystal structure of SARS-CoV-2 Mpro with 11a and 11b shows that the carbon of the aldehyde group and the catalytic site C145 of SARS-CoV-2Mpro form a standard 1.8 Å C–S covalent bond (PDB: 6LZE and 6M0K) (Figure 12B). The oxygen atom of the aldehyde group also plays a crucial role in stabilizing the conformations of the inhibitor by forming an H bond with the backbone of residue C145 in the S1’ site. The amide group on the lactam ring forms H bonds with F140 and H163. The cyclohexyl moiety of 11a at P2 deeply inserts into the S2 site and stacks with the imidazole ring of H41. The fluorine of the 3-fluorophenyl group of 11b is further stabilized by an H bond to Gln189. Relative to 11a administrated intravenously in CD-1 mice, 11b displayed a shorter T1/2 (1.65 h) and a faster clearance rate (clearance = 20.6 ml min^−1^ kg^−1^), indicating that 11a is a better candidate for further clinical study (Dai et al., 2020; Liu et al., 2020).

### Calpeptin

**FIGURE 13:**
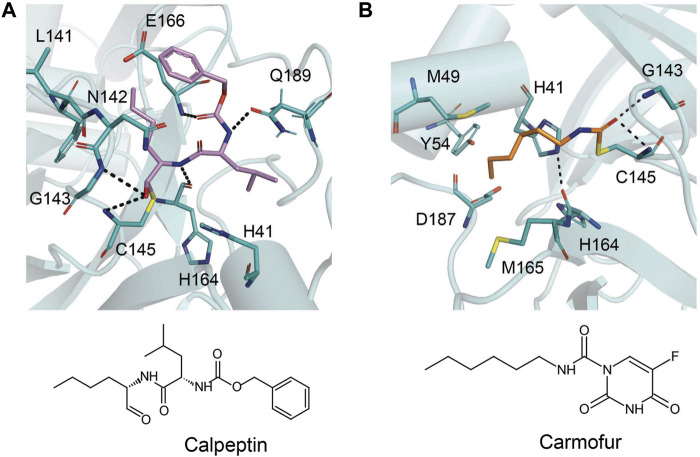
Close-up view of the interactions between SARS-CoV-2 Mpro with calpeptin and carmofur. Close-up view of interaction between SARS-CoV-2 Mpro and inhibitors in crystal structures. Mpro is shown as cartoon representation, with residues involved in the interaction shown as sticks. Inhibitors are shown as sticks. Hydrogen bonds were labeled with dashed lines. **(A)** Calpeptin (PDB: 7AKU), violet. **(B)** Carmofur (PDB: 7BUY), orange.

Calpeptin was the most potent inhibitor discovered in the large-scale X-ray crystallographic screen by Günther et al. (EC_50_ = 72 nM) (Günther et al., 2021). Calpeptin structure binds covalently *via* its aldehyde group to C145, forming a thiohemiacetal (Figure 13A). This peptidomimetic inhibitor occupies substrate pockets S1 to S3, similar to the peptidomimetic inhibitors GC-376 and N3. The peptidomimetic backbone forms H bonds to the main chain of H143, C145, H164, and E166 and the side chain of Q189 (PDB: 7AKU) (Figure 13A). The norleucine side chain of calpeptin maintains van der Waals contacts with the backbone of F140, L141, and N142 of the oxyanion hole (Günther et al., 2021).

### Carmofur

The antineoplastic drug carmofur is a derivative of 5-fluorouracil (5-FU) and has been used to treat colorectal cancer by inhibiting human acid ceramidase (Sakamoto et al., 2005). Human acid ceramidase cleaves carmofur, and the fatty acid moiety forms a covalent bond to the active site C143 (Dementiev et al., 2019). Jin et al. found carmofur as an inhibitor for SARS-CoV-2 Mpro when screening a library of about 10,000 compounds (Jin et al., 2020a). Carmofur inhibits the activity of SARS-CoV-2 Mpro *in vitro* with an IC_50_ value of 1.82 µM and inhibits viral replication with an EC_50_ value of 24.30 μM (Ma et al., 2020a). Mass spectrometry data showed that carmofur convalently binds to C145 (Jin et al., 2020a). The crystal structure of SARS-CoV-2 Mpro in complex with carmofur verifies that the compound directly modifies the catalytic cysteine and releases the 5-FU head (PDB: 7BUY) (Jin et al., 2020b). The fatty acid moiety points toward the hydrophobic S2 subsite composed of the side chains of H41, M49, Y54, M165, and D187. The inhibitor is involved in extensive hydrophilic and hydrophobic interactions with Mpro. The carbonyl oxygen of carmofur occupies the oxyanion hole and forms H bonds with the backbone amides of G143 and C145 (Figure 13B) (Jin et al., 2020b). In a study by Ma et al., the inhibition of SARS-CoV-2 Mpro by several compounds was tested for dependence on the reducing agent DTT. Carmofur could still bind in the absence of DTT but with lower potency (Ma et al., 2020a). Although carmofur is not an ideal candidate for SARS-CoV-2 Mpro, it could be further derivatized and optimized against this and future coronaviruses.

### Pelitinib

**FIGURE 14:**
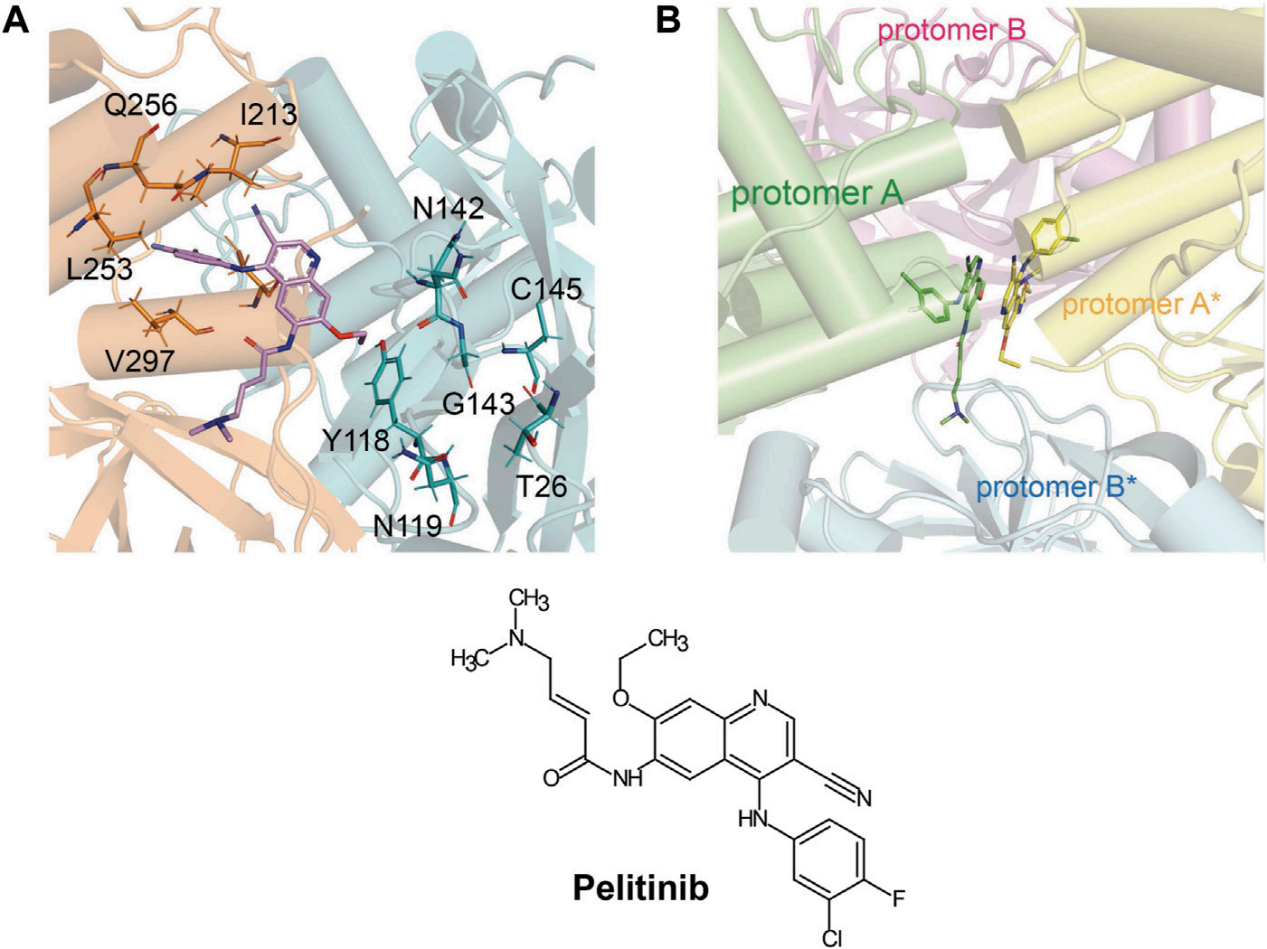
Close-up view of the interactions between SARS-CoV-2 Mpro with pelitinib. Close-up view of interaction between SARS-CoV-2 Mpro and inhibitors in crystal structures. Mpro is shown as cartoon representation, with residues involved in the interaction shown as sticks. Inhibitors are shown as sticks. Hydrogen bonds were labeled with dashed lines. **(A)** Binding site of pelitinib (PDB: 7AXM), violet, in the Mpro dimer, orange and teal. **(B)** Crystal packing of pelitinib, green and yellow sticks. One Mpro dimer is shown in green and magenta. A second dimer (*) is shown in yellow and light blue.

Pelitinib was developed as an anticancer agent to bind to a cysteine in the active site of the tyrosine kinase epidermal growth factor receptor inhibitor (Wissner et al., 2003). It shows high antiviral activity in the screen performed by Günther et al. (EC_50_ = 1.25 μM) (Günther et al., 2021). Because pelitinib is an amine-catalyzed Michael acceptor, it was predicted to target the catalytic cysteine; however, electron density map of co-crystal structure of Mpro with pelitinib shows that it binds between the two Mpro protomers (PDB: 7AXM) (Günther et al., 2021). The ethyl ether of pelitinib makes contacts with T26, N119, N142, and G143 of one protomer, which perturbs the oxyanion hole necessary for Mpro activity (Figure 14A). The aromatic moieties of pelitinib form more extensive contacts within the helical domains of the second protomer. The substituted benzyl group inserts into a hydrophobic pocket formed by residues I213, L253, Q256, V297, and C300 from domain III. The 3-cyanoquinoline moiety interacts with S301 from the end of the C-terminal helix (Günther et al., 2021). Evaluation of the crystal packing indicates that two dimers of Mpro can interact *via π*-stacking of two pelitinib molecules (Figure 14B). It remains to be seen if this oligimeric interaction occurs in solution and is therefore another mode of inhibition of this compound.

### Noncovalent Inhibitors

**FIGURE 15:**
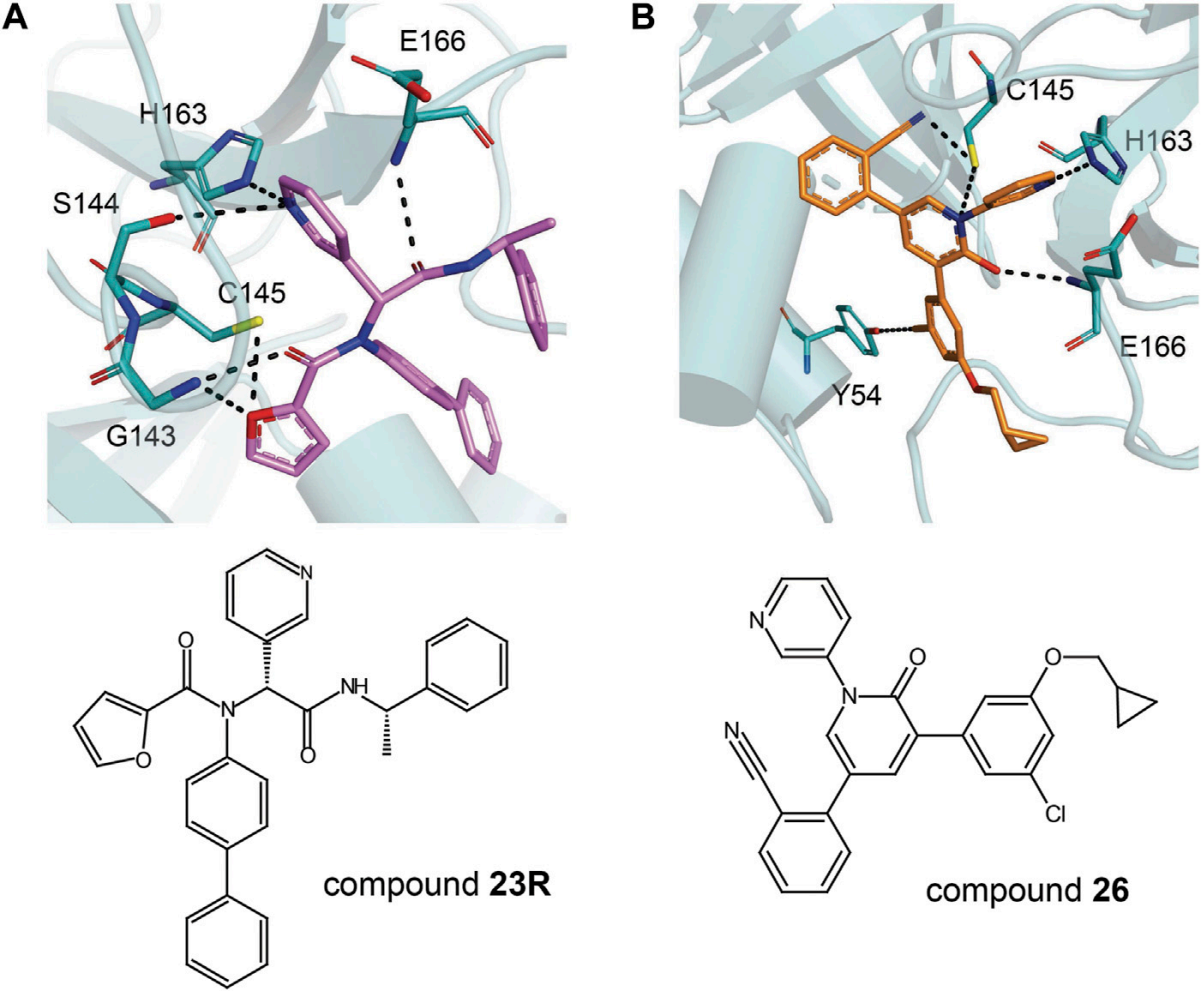
Close-up view of the interactions between SARS-CoV-2 Mpro with ML188 derivative 23R and perampanel derivative 26. Close-up view of the interactions between SARS-CoV-2 Mpro and inhibitors in crystal structures. Mpro is shown as cartoon representation, with residues involved in the interaction shown as sticks. Inhibitors are shown as violet sticks. Hydrogen bonds were labeled with dashed lines. **(A)** Compound 23R (PDB: 7KX5), violet. **(B)** Compound 26 (PDB: 7L14), orange.

Drugs acting through covalent modifications of the target may likely be associated to off-target liability and consequent potentially toxic effects (Ghosh et al., 2020). Research efforts are also devoted to the search on novel noncovalent inhibitors for 3CLpro inhibitors in order to circumvent these issues. ML188(R) is a noncovalent Mpro inhibitor derived in a high-throughput screen against SARS-CoV-1 Mpro (Jacobs et al., 2013). The pyridinyl from ML188(R) fits in the S1 pocket and forms an H bond with the H163 side chain. The furyl oxygen and its amide oxygen both form an H bond with G143. ML188(R) was reported to inhibit the SARS-CoV-1 Mpro with an IC_50_ value of 1.5 ± 0.3 μM and the SARS-CoV viral replication in Vero E6 cells with an EC_50_ value of 12.9 μM. Kitamura et al. (2021) designed and tested several noncovalent inhibitors based on ML188(R) (Kitamura et al., 2021). Compound 23 had improved enzymatic inhibition, and it was found that 23R is the active diastereomer with an IC_50_ value of 0.20 ± 0.01 μM. The antiviral activity was tested in cells expressing TMPRSS2 with an EC_50_ = 3.03 μM. The X-ray crystal structure of SARS-CoV-2 Mpro in complex with 23R reveals a ligand-induced binding pocket in between S2and S4 sites that can be explored for drug design (PDB: 7KX5) (Figure 15A). Similarly, Zhang et al. chose a weak screen hit, perampanel, to redesign due to its simple structure (Zhang et al., 2021). Free-energy calculations provided guidance for favorable modifications. Compound 26 showed effective inhibition and antiviral activity (IC_50_ = 0.170 μM and EC_50_ = 0.98 μM). The crystal structure of the compound 26 bound to Mpro shows H bonding to C145, G163, and E166, as well as halogen bonding between chlorine and Y54 (PDB: 7L14) (Figure 15B).

## Mutations in SARS-CoV-2 Papain-Like Protease and Main Protease

Mutation is a common phenomenon in viral systems and delays the identification of successful drug candidates. Constant monitoring of new variants and genetic variability within SARS-CoV-2 is extremely important for drug development and screening in order to eliminate those inhibitors with target binding sites with mutation prone residues. Genotyping of SARS-CoV-2 virus strains circulating worldwide have identified multiple recurrent non-synonymous mutations in proteases in variants of concerns (VOCs) (Table 5) (Amamuddy et al., 2020; Amin et al., 2021).

**TABLE 5:** Mutations identified in variant of concern genomes.

Variant	PLpro mutations	Mpro mutations
Alpha	A145D, M23I, T4A	—
Beta	K92N	K90R, A193V
Gamma	K232Q	—
Delta	P77L	—
Omicron	—	P132H
Eta	—	—
Iota	—	—
Kappa	T4I	—
Lambda	—	G15S
Mu	—	—
Others	P77L, T74I, T75I, D76N, K182I	G15S, A194S, L205V

**FIGURE 16:**
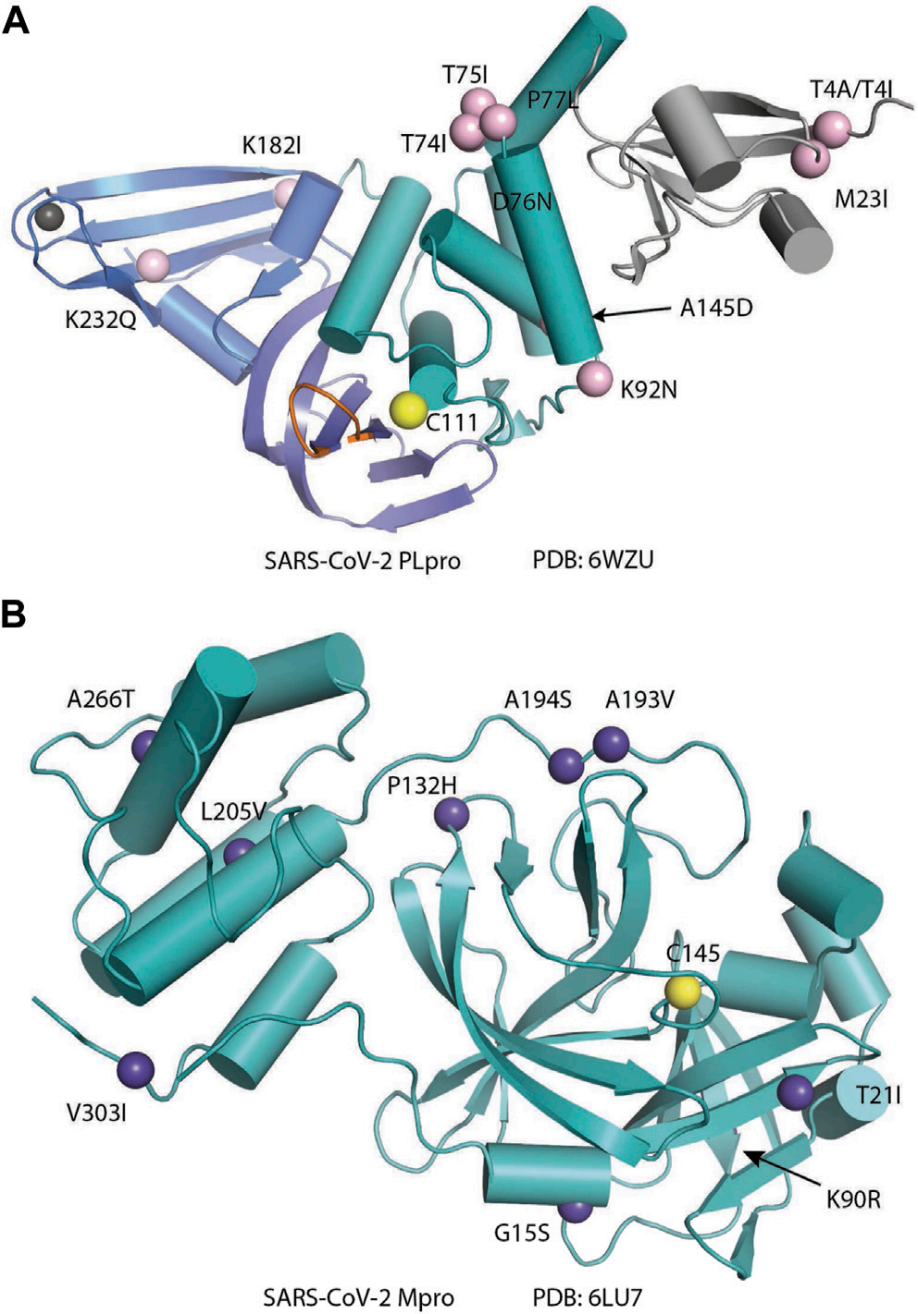
Structural mapping of mutations from variants of concern. **(A)** PLpro and **(B)** Mpro are shown as cartoon representation, with VOC mutated residues shown as spheres. The catalytic cysteines are shown as yellow spheres.

For PLpro, the mutations include A145D, M23I, and T4A from Alpha; K92N from Beta, K232Q from Gamma; P77L from Delta; T4A from Kappa; and T74I, T75I, D76N, and P77L from other stains (Figure 16A). These residues are away from catalytic site and will not disrupt the binding of inhibitors adjacent to the catalytic site; therefore, the development of PLpro inhibitors targeting P1–P4 sites is not negatively affected by the emergence of new variants. Still, the location of the mutation is related to the binding of Ubl. For example, mutation of T75 was shown to partially recover the activity of SARS-CoV-2 PLpro in cleaving K48-linked poly-Ub (Shin et al., 2020). It is interesting that multiple mutations were observed in VOCs in this region including T74, T75, N76, and P77. These mutations may potentially improve the poor reactivity of SARS-CoV-2 PLpro toward K48-linked Ub as substrate. This hypothesis and underlying mechanism are being investigated.

In SARS-CoV-2 Mpro, G15 and K90 are the most common mutations to date in VOCs (Figure 16B) (Tzou et al., 2020; Krishnamoorthy and Fakhro,2021). The mutation K90R is expected to provide stability to the domain I and improve the dimerization, which is required for enzymatic activity, and could possibly hinder compounds that target the dimer interface, such as pelitinib. Mpro appears to be relatively tolerant of mutations near the active site, and key residues in the active site (H41, F140, C145, and E166) so far show low mutation frequencies (Cross et al., 2020; Portelli et al., 2020). Mutations in key residues, such as the catalytic dyad, would produce an inactive enzyme; therefore, these mutants are not expected to evolve. However, other mutations (the C44-P52 loop, T45, S46, E47, and L50) that contribute to access to the active site have been modeled and are anticipated to be energetically favorable (Bzówka et al., 2020). Bzowka et al. recommend including P39, R40, P52, G143, G146, and/or L167 in the binding mode of Mpro inhibitors, as these are energetically unfavorable to mutate.

## Challenges in the Development of Drugs Targeting Papain-Like Protease and Main Protease

### Oxidation of Catalytic Cysteine

In an effort to obtain complex structure of SARS-CoV-1 PLpro with disulfiram, Lin et al. only observed electron density that fits beta-mercaptoethanol (PDB: 5Y3Q) (Lin et al., 2018). It seems that the catalytic cysteine of PLpro is sensitive to oxidation, and, indeed, PLpro is often purified in the presence of high concentrations of reducing reagents (Klemm et al., 2020; Shin et al., 2020; Patchett et al., 2021). Considering the WT apo structure of PLpro is scarce, whereas the structure of C111S mutant is much more abundant, high reactivity of the catalytic cysteine exhibits both an advantage for targeting it with peptide suicide inhibitors, disulfide-based inhibitors, or selenium-containing inhibitors, and it posts as a challenge that the viral protease may use an oxidation–reduction cycle to evade inhibition or simply rely on reducing reagent to remove disulfide or selenium-based inhibitors. Although WT Mpro seems to have great crystallization properties, oxidation of catalytic cysteine was also observed (Kneller et al., 2020c).

### Discrepancy in Biochemical Assay and Viral Replication Assay

Although GRL0617 is a potent inhibitor in both biochemical assays and cell-based viral replication assays, inconsistencies in some of the derivatives were reported. For example, Jerzy et al. (Osipiuk et al., 2021a) found that compounds 2 and 3 were promising PLpro inhibitors (IC_50_ values of 5.1 and 6.4 μM, respectively) but failed in the viral replication assay. Compound 5 was the weakest inhibitor *in vitro* (IC_50_ values of 32.8 μM) but performed well in the live viral replication assay (EC_50_ = 2.5 μM). Shen et al. showed XR8-89 has highest potency for PLpro inhibition (IC_50_ = 113 nM), yet it has lower EC_50_ value than XR8-23 and XR8-24 (Shen et al., 2021). It was argued that differences in cell permeability and solubility could account for the differences between the *in vitro* biochemical assay data and viral replication data. Ma et al. developed a FlipGFP assay for quantifying the intracellular PLpro inhibition, which was achievable in the biosafety level 2 (BSL-2) setting and found a positive correlation between the results from the FlipGFP-PLpro assay and the antiviral assay (Ma et al., 2021a). Whether the FlipGFP-PLpro faithfully predicts the cellular antiviral activity of PLpro inhibitors awaits further verification by others.

### Metabolic Processing

Another challenge is that some inhibitors may be easily metabolized. Báez-Santos et al. found Compound 15g being very unstable (Ghosh et al., 2010; Báez-Santos et al., 2014c). 15g has 3,4-methylenedioxy moiety, which is a known target of cytochrome P450s (Hodgson and Philpot 1974; Anders et al., 1984), whereas 3e and methoxypyridine 5c were significantly more stable (Ghosh et al., 2010; Báez-Santos et al., 2014c). Shen et al. argued that replacement of the naphthalene ring is also anticipated to improve metabolic stability (Shen et al., 2021) and found that ZN3-80 has superior stability than GRL0617 in human liver microsome stability assays (Shen et al., 2021). Cytotoxicity is also a consideration when refining these inhibitors. GRL0617, derivatives, and many other inhibitors did not show much cytotoxicity. Several inhibitors showed high selectivity toward SARS PLpro instead of DUBs, like GRL0617 (Ratia et al., 2008), compound 19 (Shan et al., 2021), and VIR250 and VIR251 (Patchett et al., 2021) (Rut et al., 2020a).

### Cell System Bias and Off-Target Inhibition

A concern for screening Mpro inhibitors is the potential for hits to have cross-reactivity with other cysteine proteases. The most likely family of off-target host proteases are the cysteine cathepsins, which are broadly expressed in many cell types and are accessible to small-molecule and peptide-based inhibitors. SARS-CoV-2 can utilize multiple pathways to enter the host cell that depend on a variety of cellular proteases among which are cathepsins B and L, TMPRSS2, and furin (Bestle et al., 2020; Hoffmann et al., 2020; Shang et al., 2020). Lead Mpro inhibitors were tested A549 + ACE2 cells with and without expression of TMPRSS2, and all inhibitors showed a loss in potency with TMPRSS2 expression suggesting that many Mpro inhibitors have some level of antiviral activity due to inhibition of cathepsin-mediated host cell entry (Steuten et al., 2021). In this case, off-target effect can potentially be studied in the scope of polypharmacology. Off-target effects may also account for the discrepancy between biochemical assay and cell-based assay for inhibitors targeting PLpro. As with any drug discovery efforts, many other aspects like membrane permeability, drug efflux, and metabolism also play a critical role in the drug development pipeline.

## Combinatorial Therapeutic Approaches

### Drug Cocktails

Another strategy to consider with protease inhibition design is the combination of strong PLpro and/or Mpro inhibitors with drugs that inhibit other viral functions or patient clearance of treatment. By inhibiting Mpro and PLpro viral proteolysis, disulfiram/ebselen can prevent efficient cleavage of the replicase polyproteins into component NSPs. In case the virus produces resistance against these proteases, disulfiram/ebselen can also inhibit the RTC core that is crucial for viral RNA synthesis, proofreading, and capping, thus restoring Remdesivir’s ability to function as a delayed chain terminator (Chen et al., 2021). In addition, the combination of GC376 and Remdesivir was shown to completely inhibit viral replication in virus plaque assay, showing an additive effect of the joint application of RdRp inhibitors and protease inhibitors targeting different viral proteins (Fu et al., 2020).
